# Cardiac adverse events associated with dual immune checkpoint inhibitors: a pharmacovigilance analysis from the FDA adverse event reporting system

**DOI:** 10.1093/ehjcvp/pvag006

**Published:** 2026-01-22

**Authors:** Huang Xia, Lei Shi, Shuimei Luo, Zequn Sun, Mengru Quan, Huazhen Xiao, Yu Chen, Jing Lin

**Affiliations:** Department of Phase I Clinical Trial Ward, Clinical Oncology School of Fujian Medical University, Fujian Cancer Hospital, Fuzhou, Fujian Province 350000, China; NHC Key Laboratory of Cancer and Metabolism (Fujian Cancer Hospital), Fuzhou, Fujian Province 350000, China; NHC Key Laboratory of Cancer and Metabolism (Fujian Cancer Hospital), Fuzhou, Fujian Province 350000, China; Department of Medical Oncology, Clinical Oncology School of Fujian Medical University, Fujian Cancer Hospital, Fuzhou, Fujian Province 350000, China; Cancer Bio-Immunotherapy Center, Clinical Oncology School of Fujian Medical University, Fujian Cancer Hospital, Fuzhou, Fujian Province 350000, China; Department of Phase I Clinical Trial Ward, Clinical Oncology School of Fujian Medical University, Fujian Cancer Hospital, Fuzhou, Fujian Province 350000, China; NHC Key Laboratory of Cancer and Metabolism (Fujian Cancer Hospital), Fuzhou, Fujian Province 350000, China; NHC Key Laboratory of Cancer and Metabolism (Fujian Cancer Hospital), Fuzhou, Fujian Province 350000, China; Department of Medical Oncology, Clinical Oncology School of Fujian Medical University, Fujian Cancer Hospital, Fuzhou, Fujian Province 350000, China; Cancer Bio-Immunotherapy Center, Clinical Oncology School of Fujian Medical University, Fujian Cancer Hospital, Fuzhou, Fujian Province 350000, China; NHC Key Laboratory of Cancer and Metabolism (Fujian Cancer Hospital), Fuzhou, Fujian Province 350000, China; Department of Medical Oncology, Clinical Oncology School of Fujian Medical University, Fujian Cancer Hospital, Fuzhou, Fujian Province 350000, China; Department of Chemistry, Fuzhou University, Fuzhou 350108, Fujian Province, China; Department of Cardiology, Shengli Clinical Medical College of Fujian Medical University, Fujian Provincial Hospital, Fuzhou University Affiliated Provincial Hospital, East Street, Fuzhou, Fujian Province 350000, China; NHC Key Laboratory of Cancer and Metabolism (Fujian Cancer Hospital), Fuzhou, Fujian Province 350000, China; Department of Medical Oncology, Clinical Oncology School of Fujian Medical University, Fujian Cancer Hospital, Fuzhou, Fujian Province 350000, China; Cancer Bio-Immunotherapy Center, Clinical Oncology School of Fujian Medical University, Fujian Cancer Hospital, Fuzhou, Fujian Province 350000, China; Department of Chemistry, Fuzhou University, Fuzhou 350108, Fujian Province, China; NHC Key Laboratory of Cancer and Metabolism (Fujian Cancer Hospital), Fuzhou, Fujian Province 350000, China; Department of Medical Oncology, Clinical Oncology School of Fujian Medical University, Fujian Cancer Hospital, Fuzhou, Fujian Province 350000, China; Cancer Bio-Immunotherapy Center, Clinical Oncology School of Fujian Medical University, Fujian Cancer Hospital, Fuzhou, Fujian Province 350000, China; Department of Chemistry, Fuzhou University, Fuzhou 350108, Fujian Province, China

**Keywords:** dual Immune checkpoint inhibitors, Immune-associated adverse events, Cardiac adverse events, FAERS database, Pharmacovigilance

## Abstract

**Aims:**

Immune-related adverse events(irAEs) are common among cancer patients receiving dual immune checkpoint inhibitors (ICI). Cardiac adverse events rank among the most severe categories of such reactions. This study aims to investigate the characteristics and influencing factors of cardiac adverse events associated with dual ICIs.

**Methods and results:**

We collected data on cardiac adverse events associated to dual ICIs from the US Food and Drug Administration Adverse Event Reporting System database (FAERS), covering the period from the first quarter of 2015 to the fourth quarter of 2024. A disproportionality analysis was performed to assess the association between different dual ICIs and cardiac adverse events, and a comprehensive study was conducted to identify potential influencing factors. Cardiac adverse events accounted for 7.5% of all ICI adverse event reports in the FAERS database. Nivolumab plus ipilimumab was associated with the highest number of significant preferred term (PT) signals, while durvalumab plus tremelimumab had the highest percentage of life-threatening outcomes. The median onset time for cardiac adverse events following dual ICIs therapy was 32 days (IQR 15–77). Myositis and myasthenia gravis were the most commonly co-reported extracardiac conditions in myocarditis cases, whereas complete atrioventricular block and cardiogenic shock were the most frequently reported intracardiac complications. Older patients, males, and those with kidney cancer were at higher risk of developing cardiac adverse events.

**Conclusion:**

This study identified distinct associations between different dual ICIs treatment strategies and various cardiac adverse events. We further identified potential risk factors and co-reported symptoms of cardiotoxicity, which may aid in the early diagnosis and monitoring of ICI-associated cardiac adverse events.

## Introduction

The advent of immune checkpoint inhibitors (ICI) has revolutionized the treatment landscape of malignancies, achieving remarkable success across various cancer types.^1^ Programmed cell death-1/programmed death-ligand 1 (PD-1/PD-L1) and cytotoxic T lymphocyte-associated antigen-4 (CTLA-4) are the most commonly ICIs. Compared to ICI monotherapy, dual ICIs (combination ICI immunotherapy with anti-PD (L)1 and anti-CTLA4) demonstrate longer progression-free survival (PFS) and overall survival (OS) outcomes.^2^ In patients with unresectable or metastatic melanoma, the PFS and objective response rate (ORR) were 11.5 months and 57.6% with the combination of anti-PD-1 and anti-CTLA-4, compared with 6.5 months and 43.7% with anti-PD-1alone, and 2.9 months and 19% with anti-CTLA-4 alone.^3^ In advanced renal cell carcinoma, dual ICIs therapy achieved an ORR of 42%, including 9% complete responses, with a PFS of 11.6 months and an 18-month OS rate of 75%. Compared with sunitinib, a standard first-line treatment for renal cell carcinoma, the risk of disease progression or death was significantly reduced (HR, 0.63; *P* < 0.001).^4^ Currently, dual ICIs have been employed in the treatment of various malignancies, including melanoma, renal cell carcinoma, colorectal cancer, non-small cell lung cancer, and hepatocellular carcinoma.

However, mounting evidence shows that dual ICIs cause significantly higher incidence and severity of immune-related adverse events (irAEs) than ICI monotherapy,^5,6^. In a retrospective pharmacovigilance study, combination therapy with nivolumab (anti-PD-1) and ipilimumab (anti-CTLA-4) increased the risk of myocarditis by 4.74-fold compared to nivolumab alone.^7^ In another study, the overall prevalence of irAEs (over 90%) and their severity (with grade ≥3 adverse events accounting for approximately 60% of all irAEs were higher with the combination of anti-CTLA-4 and anti-PD-1 or anti-PD-L1 than with monotherapy).^8^ In addition, different ICIs are associated with distinct irAE spectra, which may be driven by variations in the immune microenvironment that underlie tissue-specific patterns of immune activation.^9^ Dual ICI therapy can further enhance the expansion of CD4^+^ and CD8^+^ T cells within the anti-CTLA-4 induced immune microenvironment and reduce the proportion of regulatory T cells in the tumour, a phenomenon that is not observed with monotherapy.^10^

IrAEs can involve multiple organs, usually occurring within weeks to months after treatment initiation, though delayed onset may also occur. They are often more severe than conventional autoimmune diseases and may lead to treatment interruption. Effective management and personalized strategies are essential to reduce morbidity and mortality.

Among reported irAEs, cardiac toxicities have emerged as a growing concern. Although less common than gastrointestinal (8–27%) or dermatologic toxicities (up to 72%), ICI-related cardiotoxicities are often life-threatening.^11^ Previously identified ICI-associated cardiac adverse events include myocarditis, cardiomyopathy, pericarditis, acute coronary syndrome, arrhythmias, and cardiac failure.^12,13^ In particular, myocarditis is the most lethal, with a reported mortality rate of 30–50%.^14^ Moreover, myocarditis associated with dual ICI therapy occurs at a higher risk and is frequently accompanied by overlapping myositis and myasthenia gravis.^7^

Several pharmacovigilance analyses have investigated the cardiac adverse events of ICI monotherapy.^15,16^ However, data focusing specifically on dual immunotherapy-associated cardiac adverse events remain limited. Therefore, in this retrospective observational pharmacovigilance study, we aimed to comprehensively evaluate dual ICIs-associated cardiac adverse events using disproportionality analysis based on real-world data from the U.S. Food and Drug Administration Adverse Event Reporting System database (FAERS), accompanied by a detailed characterization of the clinical features of affected patients and identification of susceptible populations and high-risk groups for serious cardiac adverse events outcomes.

## Materials and methods

### Data source

This retrospective, observational pharmacovigilance study was conducted based on the FAERS database. We mainly focused on three dual ICI strategies: pembrolizumab plus ipilimumab, durvalumab plus tremelimumab, and nivolumab plus ipilimumab.Monotherapy was defined as treatment with a single class of ICIs—anti–PD-(L)1 or anti–CTLA-4—excluding cases with concurrent checkpoint inhibitor use. The included agents were anti–PD-1 (cemiplimab, nivolumab, pembrolizumab), anti–PD-L1 (atezolizumab, avelumab, durvalumab), and anti–CTLA-4 (ipilimumab, tremelimumab). These ICIs served as keywords to extract relevant case reports from the database, covering the period from 1 January 2015, to 30 December 2024. which is available at: https://fis.fda.gov/extensions/FPD-QDE-FAERS/FPD-QDE-FAERS.html.

### Classification and extraction of adverse reactions

Adverse reactions reported in the FAERS database are entered using preferred term (PT) codes from the Medical Dictionary for Regulatory Activities (MedDRA). The PTs provide distinct descriptors for an individual’s medical concepts, such as symptoms, side effects, and disease diagnosis. Different PTs are grouped into various system organ classes (SOCs). According to the SOC classification ‘Cardiac disorders’ in MedDRA version 27.1, all PTs related to cardiac adverse events, as well as cardiac injury biomarkers and electrocardiogram abnormalities, were extracted and included in the subsequent analysis.

### Data processing procedure

**Figure 1:**
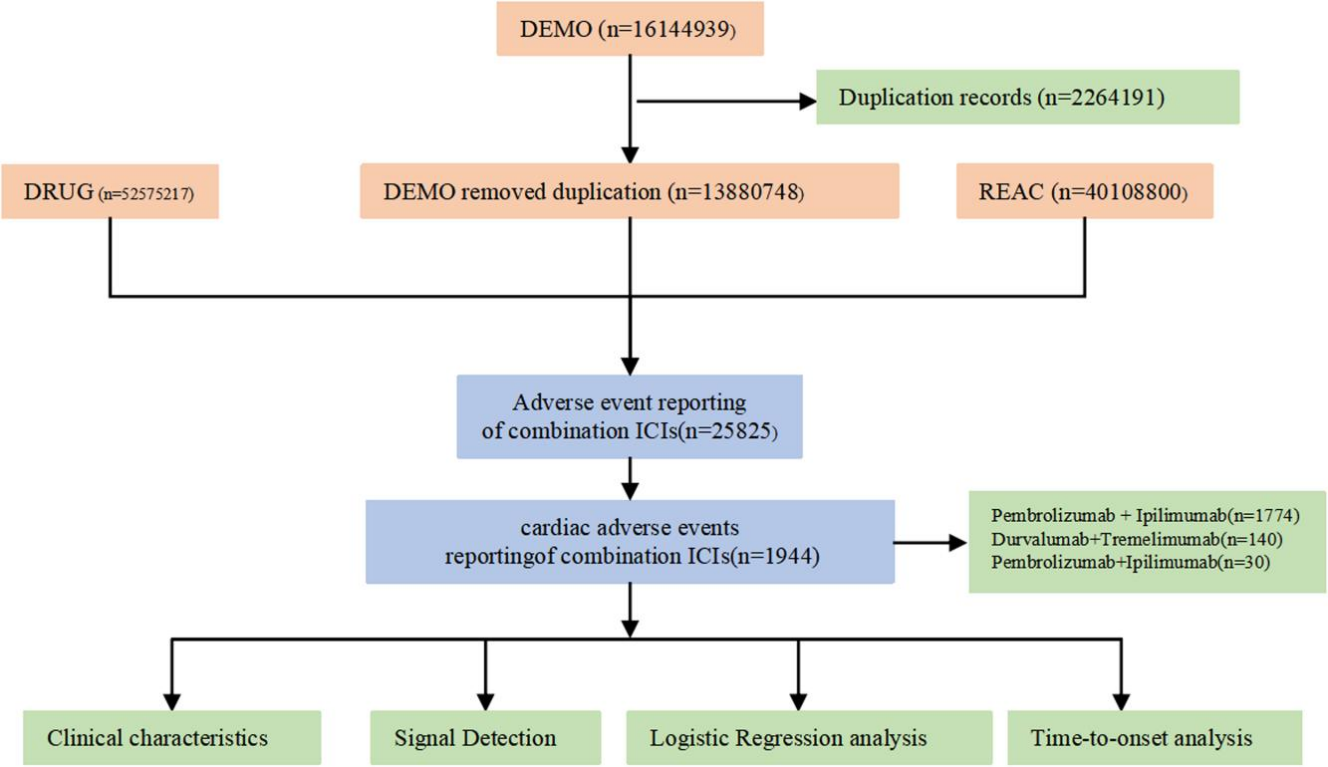
Flow chart showing the analysis process of the study: detailed description of the selection process of cardiac adverse events for dual immune checkpoint inhibitors (ICIs) in the Food and Drug Administration Adverse Event Reporting System (FAERS).

Duplicate reports were processed according to FDA recommendations. For records with the same CASEID, the one with the most recent FDA received date (FDA_DT) was retained; if both CASEID and FDA_DT were identical, the report with the highest PRIMARYID was kept.^17^ For dual ICI combinations, cases were included only when at least one agent was designated as the primary suspect drug. A total of 25 852 patients who received Dual ICIs were identified, among whom 1944 experienced cardiac adverse events (*Figure 1*). ICI-associated cardiac adverse events were defined as cardiac events reported in patients who received dual ICI therapy in the FAERS database, where at least one immune checkpoint inhibitor was designated as the primary suspect drug (see Supplementary material online, *Table S1*). Adverse events were coded using spontaneously reported PTs, including myocarditis and immune-mediated myocarditis. Although immune-mediated myocarditis represents a specific subtype of myocarditis, direct reporting of this PT may reflect explicit attribution of an immune-related aetiology by the reporter. Therefore, these PTs were analysed and presented separately.

### Disproportionality analysis

Disproportionality analysis is commonly used in pharmacovigilance to evaluate associations between drugs and adverse events. In this study, four methods—reporting odds ratio (ROR),^18^ proportional reporting ratio, Bayesian Confidence Propagation Neural Network, and Empirical Bayes Geometric Mean—were employed to assess the potential association between dual ICIs and adverse events (AEs).^19^ These methods were calculated based on the disproportionality contingency table (see Supplementary material online, *Table S2*), with the corresponding signal values summarized in Supplementary material online, *Table S3*.

### Statistical analysis

For categorical variables, we used the chi-square test or Fisher’s exact test, depending on the sample size and the expected frequencies in contingency tables. As time-to-onset of cardiac adverse events, the continuous variables in our study we analysed using the Kruskal–Wallis test for their analysis. Univariate and multivariate logistic regression analyses were used to calculate odds ratios (ORs) for cardiac adverse events. fatal events’ were defined as adverse event reports with outcomes of either death or life-threatening. All statistical procedures were conducted using R software (version 4.3.1).

## Results

### Descriptive analysis of cardiac adverse events in patients receiving dual ICI therapy

From January 2015 to December 2024, we identified 1944 reports of cardiac adverse events from the FAERS database and summarized the clinical characteristics of these patients. Among these reports, the proportion of female patients (539, 27.7%) was lower than that of male patients (1174, 60.4%). The age group with the most reports was >65 years (996, 51.2%). Among the dual ICIs regimens, nivolumab plus ipilimumab was the most frequently used (177 4,91.2%). Reports mainly came from physicians (898,46.2%). Most of the reports originated from the USA (632,32.5%) and Japan (459,23.6%), cases with indications for melanoma (581,29.9%), lung cancer (366,18.8%), and kidney cancer (230,11.8%) accounted for the majority (*Table 1*).

**Table 1:** Patients characteristics

Characteristics	No. of Patients (%)
Gender	
Male	1174(60.4%)
Female	539(27.7%).
Miss	231(11.9%)
Age	
<18	13(0.7%)
18–65	564(29.0%)
>65	996(51.2%)
Miss	371(19.1%)
Treatment strategy	
Nivolumab + Ipilimumab	1774(91.2%)
Durvalumab + Tremelimumab	140(7.2%)
Pembrolizumab + Ipilimumab	30(1.5%)
TOP 3 Reported Country	
United States	632(32.5%)
Japan	459(23.6%)
France	193(9.9%)
TOP 3 indications	
Melanoma	581(29.9%)
Lung cancer	366(18.8%)
kidney cancer	230(11.8%)
Reporter	
Consumer	139(7.2%)
Physicians	898(46.2%)
Pharmacist	86(4.4%)
Other healthcare professional	812(41.7%)
Miss	9(0.5%)

### Cardiotoxicity among dual ICIs users in the FDA adverse events reporting system, 2015–2024

**Figure 2:**
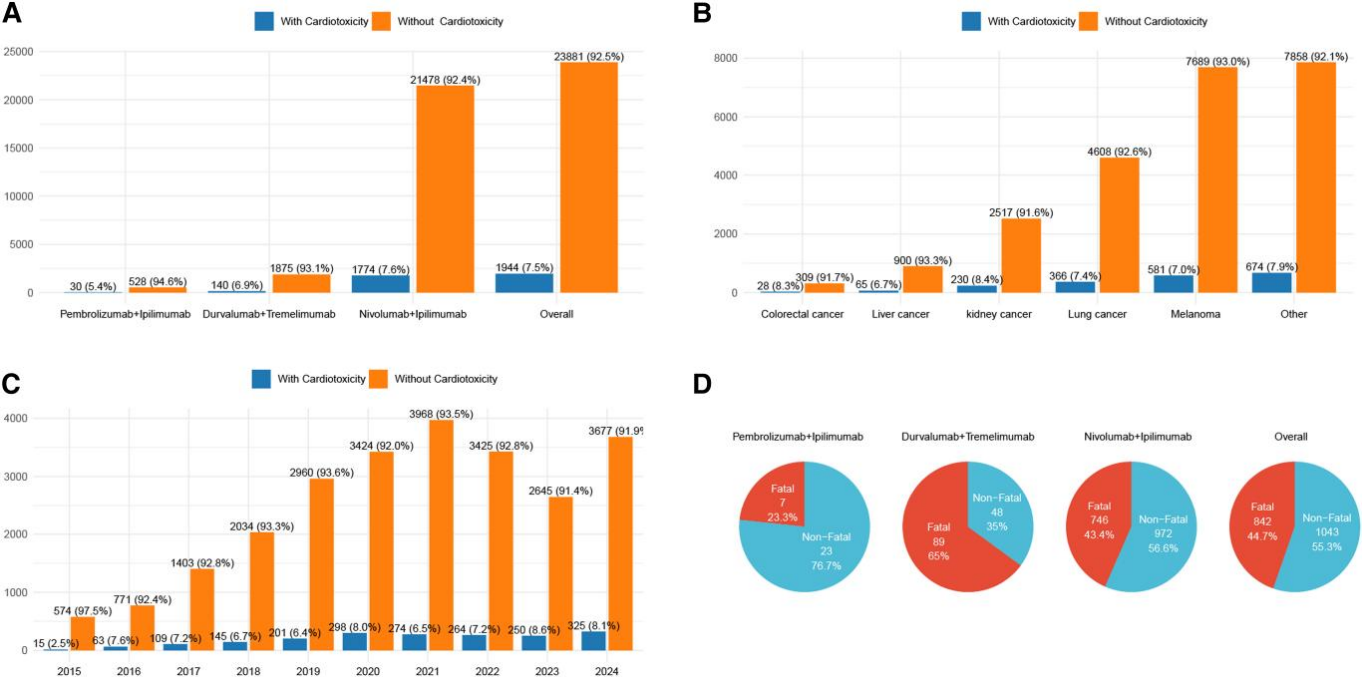
Statistics on the occurrence of cardiac adverse events in dual ICIs reports from the FAERS database during 2015–2024. *A*) The number of reports with and without cardiac adverse events associated with different dual ICI treatment strategies in the FAERS database from 2014 to 2024, along with overall report counts. *B*) The number of reports with or without cardiac adverse events among patients with different primary tumors in the FAERS database from 2014 to 2024. *C*) The number of dual ICI reports with and without cardiac adverse events in the FAERS database from 2014 to 2024, along with the overall number of reports. *D*) The percentage of fatal and non-fatal outcomes associated with different dual ICI treatment strategies.

Cardiac adverse events accounted for only a small fraction of the total adverse reactions in all of the dual ICI reports, with a case number of 1944, which accounted for 7.5% (1944/25825) of the overall cases. Among patients receiving monotherapy with ICIs, cardiac adverse events accounted for 6.2% (8583/138877) of all reported adverse events. Specifically, the proportions were 6.5% (6375/98599) for anti–PD-1, 5.9% (2005/33881) for anti–PD-L1, and 3.2% (203/6397) for anti–CTLA-4 (see Supplementary material online, *Figure S3B*). In addition, the occurrence of cardiac adverse events did not differ among the various dual ICIs treatment strategies. Specifically, the proportion of cases with cardiac adverse events was 5.4% among patients receiving pembrolizumab plus ipilimumab, 6.9% among those treated with durvalumab plus tremelimumab, and 7.6% among those receiving nivolumab plus ipilimumab (*Figure 2A*). The occurrence of cardiac adverse events differed significantly among patients with different cancer types. The proportion of cases with cardiac adverse events was 8.4% in kidney cancer, 8.3% in colorectal cancer, 7.4% in lung cancer, 7.0% in melanoma, and 6.7% in liver cancer (*Figure 2B*). In terms of yearly distribution, the number of cases in 2015 accounted for the smallest proportion (2.5%) of the total reported that year. From 2016 to 2024, the annual number of cardiac adverse event cases accounted for a relatively stable proportion (6.4–8.6%) of the total reported cases each year (*Figure 2C*).

In cases where cardiac adverse events occurred, more than 40% resulted in a fatal outcome. Although cardiac adverse events may not be the direct cause of fatal outcome, analysing the differences between potentially fatal and non-fatal cases may provide insights into improving patient outcomes. The proportion of fatal outcomes associated with dual ICIs (44.2%, 842/1905) was slightly higher than that observed with monotherapy (40.6%, 3315/8174). Among monotherapy cases, the fatal rates were 41.5% (2535/6106) for anti–PD-1 48.8% (681/1396) for anti–PD-L1, and 48.8% (99/203) for anti–CTLA-4 (see Supplementary material online, *Figure S3A*).

The distribution of fatal and non-fatal cardiac adverse events significantly differed among the three dual ICIs treatment strategies (*P* < 0.01). Among patients treated with pembrolizumab plus ipilimumab, the fatality rate was 23.3% (7/30). For durvalumab plus tremelimumab, the fatality rate was 65.0% (89/137), while for nivolumab plus ipilimumab, the fatality rate was 43.4% (746/1718) (*Figure 2D*). Among patients receiving dual ICIs therapy. The proportion of fatal events was higher in the older age group (≥65 years, 46.9%, 456/972) compared to the younger age group (18–65 years, 40.9%, 222/542). No significant difference in the proportion of fatal events was observed between male (45.9%, 521/1135) and female (42.9%, 225/525) patients.

### Scanning for combination ICI-related cardiac adverse events

**Figure 3:**
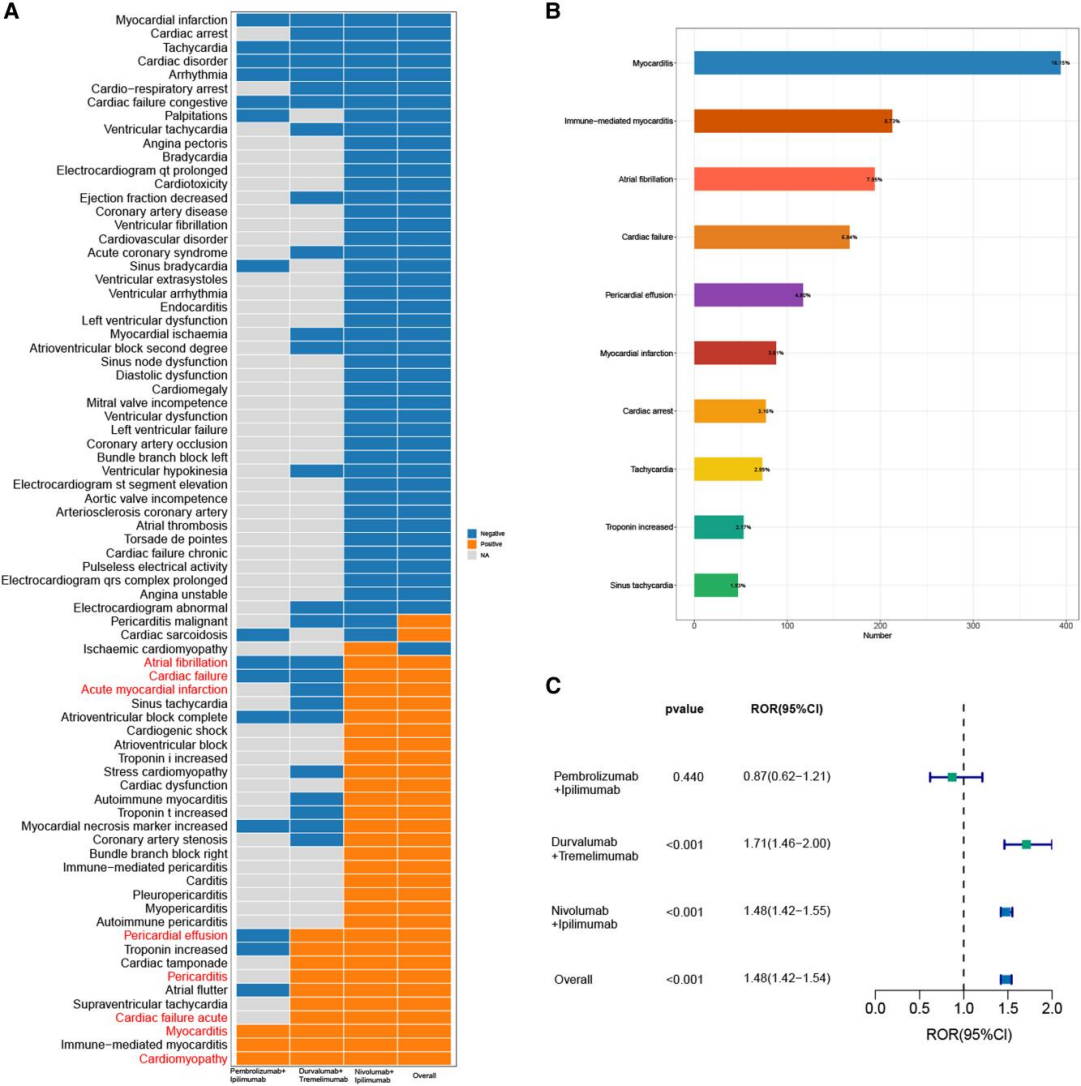
Scanning for adverse cardiac adverse events based on the FAERS database. *A*) Heatmap showing the disproportionality analysis signals for 74 cardiac adverse events with at least 3 reported cases in the FAERS database under different dual ICI treatment strategies (including overall, pembrolizumab plus ipilimumab, durvalumab plus tremelimumab, and nivolumab plus ipilimumab). Gray indicates no reported cases, orange indicates a positive signal (associated), and blue indicates no signal (not associated). *B*) Bar chart showing the top 10 most frequently reported cardiac adverse events associated with dual ICI therapy. *C*) Forest plot showing the reporting odds ratios (RORs) for cardiac adverse events associated with different dual ICI treatment strategies.

The most common cardiac adverse events (PTs) associated with dual ICIs were myocarditis [myocarditis: (394, 16.15%), mortality:38.3%, immune-mediated myocarditis: (213, 8.73%), mortality:28.17%], atrial fibrillation (194, 7.95%. mortality:20.67%), cardiac failure (167, 6.84%. mortality:32,3%), pericardial effusion (117, 4.80%. mortality:15.38%), and myocardial infarction (88, 3.61%. mortality:42.5%) (*Figure 3B*, Supplementary material online, *table S4*).

In the data mining process, the entire FAERS database served as the control. In the disproportionality analysis of cardiac adverse events, the ROR was used as the primary algorithm for detecting disproportionality signals of cardiac adverse events, while the other three algorithms served as complementary validation algorithms. On the whole, dual ICIs are significantly associated with the occurrence of cardiac adverse events [ROR 1.48 (95%Cl: 1.42–1.54)]. In addition, durvalumab plus tremelimumab was significantly associated with the occurrence of cardiac adverse events and showed the highest ROR [ROR 1.71(95%Cl: 1.46–2.00)]. Pembrolizumab plus ipilimumab did not demonstrate a significant association with cardiac adverse event [ROR 0.87(95%CI: 0.62–1.21)]; however, in our further analysis, they were significantly associated with some specific cardiac adverse events, revealing markedly strong signals. (*Figure 3C*).

We identified distinct associations between various cardiac adverse events and different combination strategies of ICIs (*Figure 3A*, Supplementary material online, *table S5*-*S8*). Pembrolizumab plus ipilimumab, durvalumab plus tremelimumab, and nivolumab plus ipilimumab were associated with a significant risk signal for cardiomyopathy [ROR 8.47 (95%Cl: 2.73–26.31), 6.02 (95% Cl: 2.5–14.48), 2.75 (95% Cl: 1.98–3.82)], respectively). Pembrolizumab plus ipilimumab, durvalumab plus tremelimumab, and nivolumab plus ipilimumab were associated with a significant myocarditis risk signal [ROR 14.63 (95%Cl: 6.08–35.19), 50.48 (95%Cl: 36.94–68.97), 28.93 (95%Cl: 25.98–32.22)], respectively. A statistically significant association with pericarditis involvement was observed for both durvalumab plus tremelimumab and nivolumab plus ipilimumab [ROR 5.05 (95%Cl: 2.27–11.24), 1.81 (95%Cl: 1.29–2.54), respectively], whereas no such cases were reported for pembrolizumab plus ipilimumab. A statistically significant association with cardiac failure acute involvement was observed for both durvalumab plus tremelimumab and nivolumab plus ipilimumab [ROR 6.34 (95%Cl: 2.04–19.69), 2.28 (95%Cl: 1.42–3.67), respectively]. Risk signals for atrial fibrillation and acute myocardial infarction were associated only with the nivolumab plus ipilimumab [ROR 1.83 (95%Cl: 1.59–2.12), 1.58 (95%Cl: 1.17–2.13), respectively).

To further explore potential differences across tumour types, subgroup analyses were performed for patients with melanoma, kidney cancer, and lung cancer who received dual ICIs therapy. In patients with melanoma, the most frequently reported cardiac adverse events were myocarditis [myocarditis: (133, 17.66%), immune-mediated myocarditis: (64, 8.50%)], atrial fibrillation (69, 9.16%), cardiac failure (33, 4.38%), pericardial effusion (32, 4.25%), and myocardial infarction (30, 4.00%). In lung cancer patients, the most commonly reported cardiac adverse events included myocarditis [myocarditis: (53, 11.16%), immune-mediated myocarditis: (24, 5.05%)], cardiac failure (43, 9.05%), pericardial effusion (35, 7.37%), atrial fibrillation (33, 6.95%), and cardiac tamponade (19, 4.00%). Among patients with kidney cancer, the most frequently reported cardiac adverse events were myocarditis [myocarditis: (55, 20.30%), immune-mediated myocarditis: (17, 6.27%)), atrial fibrillation (24, 8.86%), cardiac failure (18, 6.64%), pericardial effusion (18, 6.64%), cardiac arrest (14, 5.67%), and acute myocardial infarction (10, 3.69%).

Disproportionality analyses were further conducted for the three cancer subgroups—melanoma, lung cancer, and kidney cancer (see Supplementary material online, *table S9*-*S11*). For myocarditis and atrial fibrillation, kidney cancer patients exhibited the highest RORs. For cardiac failure and pericardial effusion, the ROR was highest in lung cancer patients, whereas melanoma patients showed a lower signal for cardiac failure.

### Time to onset analysis

**Figure 4:**
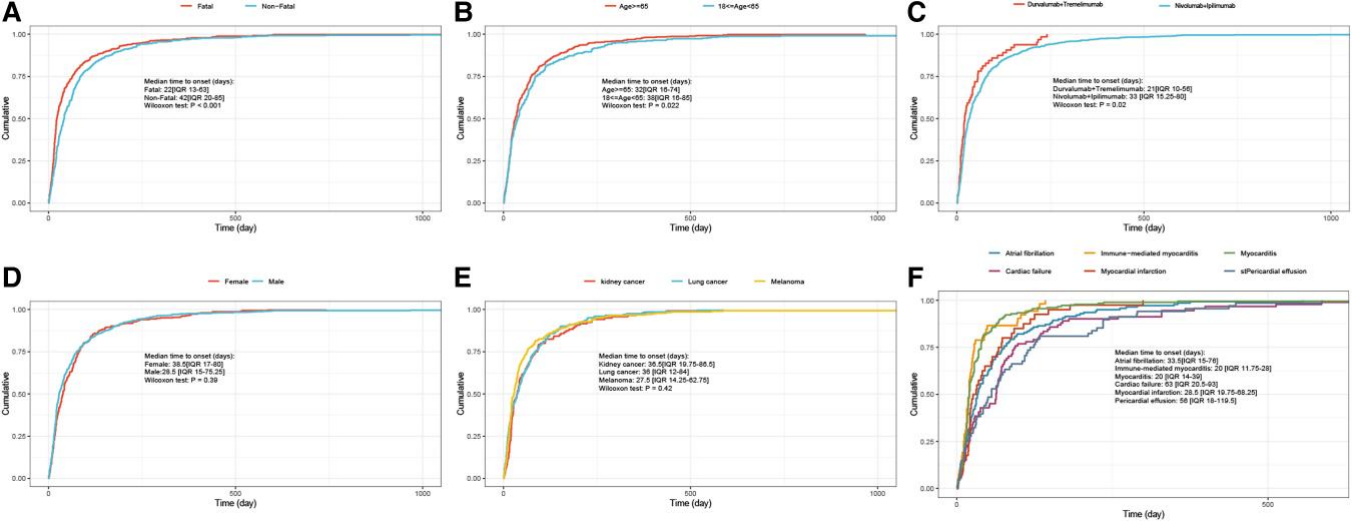
Time to onset of cardiac adverse events. A, B, C, D and *E*) Cumulative distribution curves showing the time to onset of cardiac adverse events following dual ICI therapy across different subgroups (fatal vs. non-fatal outcomes, age groups, treatment strategies, gender, indications). *F*) Cumulative distribution curves depicting time to onset of common cardiac adverse events.

More than 60% of cardiac adverse events occurred during the first 2 months of initiating dual ICIs treatments, with a median onset time of 32 days (IQR 15–77), The median onset time was significantly shorter in the fatal group than in the non-fatal group (Days: 22 vs. 42, *P* < 0.001) (*Figure 4A*). The median onset time was significantly shorter in the >65 age group compared to the 18–65 age group (Days: 32 vs. 38, *P* = 0.02) (*Figure 4B*). Different dual ICIs treatment strategies may influence the onset time of cardiac adverse events (*P* = 0.02) (*Figure 4C*). The median onset time of durvalumab plus tremelimumab was 21days (IQR 10–56). The median onset time of nivolumab plus Ipilimumab was 33 days (IQR 15.25–80). In addition, we compared the median time to onset of cardiac adverse events between dual and monotherapy. The median onset time was earlier in patients receiving dual ICI therapy than in those receiving monotherapy. The median time to onset was 32 days (IQR 15–77) for dual ICI therapy and 41days (IQR 17–113.75) for monotherapy. For specific agents, the median time to onset was 40 days (IQR 17–112) for anti–PD-1, 42 days (IQR 16–121) for anti–PD-L1 and 48.5 days (IQR 24.75–91) for anti–CTLA-4 (see Supplementary material online, *Figure S3C*). We did not observe that gender and indications had a significant impact on the onset time of cardiac adverse events (*Figure 4D, E*).

We further analysed the median time to onset of different cardiovascular events following dual ICI therapy (see Supplementary material online, *table S12*).The median time to onset for several common cardiovascular events was as follows: atrial fibrillation, 33.5 days (IQR 15–76); immune-mediated myocarditis, 20 days (IQR 11.75–28); myocarditis, 20 days (IQR 14–39); cardiac failure, 63 days (IQR 20.5–93); myocardial infarction, 28.5 days (IQR 19.75–68.25); and pericardial effusion, 56 days (IQR 18–119.5) (*Figure 4F*).

### Co-reported events and influencing factors for cardiac adverse events

**Figure 5:**
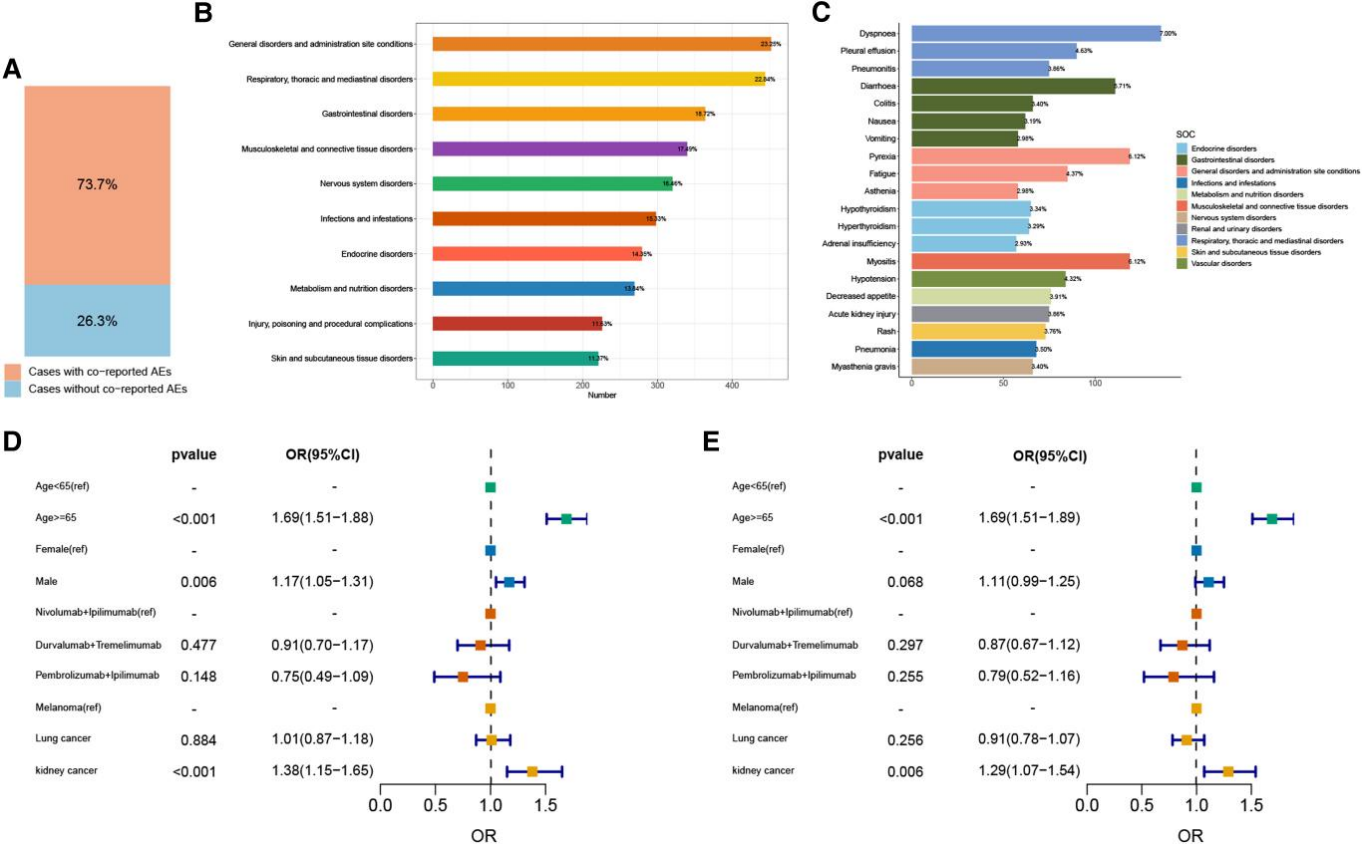
Co-reported events and influencing factors for cardiac adverse events. *A*) Bar plot shows the proportion of cases with and without co-reported adverse events incases with cardiac adverse events. *B*) The bar chart displays the most common co-reported adverse events by SOC level. The percentages marked in the chart indicate the proportion of cases with a particular adverse event relative to the total number of cardiac adverse event cases. *C*) The bar chart displays the statistical data for the most common co-reported adverse event PTs. The colors represent the corresponding SOC for each PT. The percentages marked in the chart indicate the proportion of cases with a particular adverse event relative to the total number of cardiac adverse event cases. *D*)The forest plot displays the results of univariate logistic regression analysis on factors influencing cardiac adverse events. *E*) The forest plot displays the results of multivariate logistic regression analysis on factors influencing cardiac adverse events.

We have additionally examined co-reported adverse events that may act as influencing factors for cardiac adverse events. Among the 1944 cases with cardiac adverse events that we screened, 73.7% of cases were accompanied by the occurrence of other adverse events, while 26.3% of cases had only cardiac adverse events (*Figure 5A*). Among cases with other concomitant adverse events, general disorders, respiratory, thoracic and mediastinal disorders, gastrointestinal disorders, musculoskeletal and connective tissue disorders, and nervous system disorders, the top five most frequently reported concomitant adverse events, which occurred in more than 15% of cardiac adverse events (*Figure 5B*). Additionally, dyspnoea (7.0%), myositis (6.12%), pyrexia (6.12%), diarrhoea (5.71%), and pleural effusion (4.63%), which were among the top 5 of co-reported adverse events (*Figure 5C*). As myocarditis (including PTs: ‘myocarditis’ and ‘immune-mediated myocarditis’) was the most frequently reported and one of the most severe cardiac adverse events, we further analysed its co-reported adverse events. Approximately 77.6% of myocarditis patients experienced co-reported adverse events (see Supplementary material online, *Figure S1A*). Myositis and myasthenia gravis were the most co-reported (see Supplementary material online, *Figure S1C*). Specifically, about 8.3% of patients presented with both myositis and myasthenia gravis, 15.3% with myositis alone, and 4.3% with myasthenia gravis alone (see Supplementary material online, *Figure S1B*). Complete atrioventricular block (5.27%) and cardiogenic shock (3.67%) were the most commonly co-reported intracardiac complications (see Supplementary material online, *Figure S1C*).

Among patients who developed atrial fibrillation after dual ICIs therapy, 81% had co-reported adverse events (see Supplementary material online, *Figure S2A*). The most frequently co-reported were diarrhoea (11.86%), hyperthyroidism (11.34%), pneumonia (7.22%), colitis (6.70%), and dyspnoea (6.70%) (see Supplementary material online, *Figure S2B*). In patients who developed myocardial infarction following dual ICI therapy, 72.8% had co-reported adverse event (see Supplementary material online, *Figure S2C*). The most common co-reported conditions included acute kidney injury (5.15%), myocarditis (5.15%), pyrexia (5.15%), sepsis (5.15%), asthenia (4.41%), and diarrhoea (4.41%) (see Supplementary material online, *Figure S2D*). Among patients who developed cardiac failure after dual ICIs therapy, 80.7% had co-reported adverse events (see Supplementary material online, *Figure S2E*). The most frequently co-reported conditions were immune-mediated myocarditis (10.53%), acute kidney injury (7.02%), diarrhoea (7.02%), dyspnoea (7.02%), hyponatremia (7.02%), and respiratory failure (7.02%) (see Supplementary material online, *Figure S2F*).

We further explored factors that might influence the occurrence of cardiac adverse events by univariate and multivariate logistic regression analyses based on the total reports involving dual ICIs. The type of dual ICIs was not identified as an independent influencing factor for the occurrence of cardiac adverse events. Age ≥65 years was associated with a higher risk of cardiac adverse events compared to the 18–65 age group [univariate OR: 1.69 (1.51–1.88), *P* < 0.001, multivariate OR: 1.69 (1.51–1.89), *P* < 0.001]. Male was associated with a higher risk compared to the female [univariate OR: 1.17 (1.05–1.31), *P* = 0.006, multivariate OR: 1.11 (0.99–1.25), *P* = 0.068]. In the FAERS database, the most frequently reported cardiac adverse events following dual ICI therapy were observed in patients with melanoma, lung cancer, and kidney cancer. Among them, kidney cancer patients [univariate OR: 1.38 (1.15–1.65), *P* < 0.001), multivariate OR: 1.29 (1.07–1.54), *P* = 0.006] had a higher risk of developing cardiac adverse events compared to melanoma patients (*Figure 5D, E*).

## Discussion

Over the past decade, the advent of ICIs has profoundly transformed the landscape of cancer treatment. An increasing number of cancer patients have received treatment with ICIs and derived benefits from it. While ICIs activate the immune system to fight tumours, they can also trigger irAEs that affect multiple organ systems, among which cardiac adverse events are among the most severe. Previous studies have extensively evaluated the cardiovascular safety profile of ICI monotherapies,^16^ systematic studies on cardiac adverse events caused by dual ICI strategies remain limited. Dual ICIs therapy is currently approved for multiple cancer indications and has demonstrated superior efficacy compared with single-agent ICI therapy.^20,21^ its safety profile and limitations warrant careful consideration. Therefore, this study specifically focuses on characterizing cardiac adverse events induced by dual ICIs.

Our research provided a comprehensive spectrum of cardiac adverse events induced by different dual ICIs, precisely presenting indicators of cardiac adverse events in different dual ICIs Myocarditis, atrial fibrillation, cardiac failure, pericardial effusion, and myocardial infarction were the most commonly reported cardiac adverse events. This is consistent with previous reports.^22,23^ Risk signals for cardiomyopathy and myocarditis were detected across all three dual ICIs strategies. However, current clinical and research focus emphasizes myocarditis, these results indicate that cardiomyopathy, although less acutely dramatic, deserves closer monitoring and further investigation, particularly in the setting of dual ICIs. Although only two cases of cardiac failure and no cases of pericarditis were reported for pembrolizumab plus ipilimumab. This may be due to the relatively limited clinical use of the combination therapy of pembrolizumab and ipilimumab, resulting in insufficient data in the FAERS database. Therefore, caution and continued pharmacovigilance remain essential when interpreting these findings. Interestingly, although myocardial infarction was among the most frequently reported cardiac adverse events associated with dual ICI therapy, no significant disproportionality signal was detected [ROR:0.6 (95%Cl: 0.49–0.75)]. However, a prospective study has shown that the use of immune checkpoint inhibitors increases the incidence of myocardial infarction.^24^ This suggests that the real-world incidence of immune checkpoint inhibitor-associated myocardial infarction may be underestimated, possibly due to its nonspecific symptoms, low incidence, and incomplete diagnostic evaluation.

In further investigations of factors influencing cardiac adverse events associated with dual immune checkpoint inhibitor therapy, we observed that patients over 65 years of age exhibited a higher incidence and earlier onset of such events. This is akin to findings from some previous studies, which indicated that the elderly population experiences a higher incidence of cardiac toxicity following ICIs.^25,26^ However, some studies have suggested that age is not a significant risk factor for cardiac adverse events.^27^ It is undeniable that cardiovascular diseases and their predisposing factors are inherently prevalent in the elderly population, often accompanied by a relatively poor prognosis. This may contribute to the increased occurrence of cardiac adverse events in the elderly following ICI treatment. Although it is sometimes difficult to distinguish whether cardiac-related events in real-world settings are caused by ICI or by pre-existing conditions, early identification and intervention remain essential to improving outcomes in elderly patients.

In this study, gender has been identified as an influencing factor for the occurrence of cardiac adverse events, with males exhibiting a slightly elevated risk compared to females. Some early studies suggested that there was no association between sex and the occurrence of irAEs.^28^ However, recent studies have suggested that the types of IrAEs may differ between sexes, with females predominantly experiencing endocrine-related adverse events.^29^ A meta-analysis has indicated that the male population carries a higher risk of cardiovascular toxicity.^30^ Another retrospective analysis has shown that females are more likely to experience cardiac adverse events.^31^ Although the impact of gender on cardiac adverse reactions has not yet reached a definitive conclusion at present, the aforementioned studies suggest that in the era of immunotherapy, attention should be paid to the influence of gender on immunotherapy-related cardiac adverse events.

In terms of ICI treatment strategies, the combination of nivolumab plus ipilimumab accounted for the highest number of reported adverse events, followed by durvalumab plus tremelimumab, which may indirectly reflect the more frequent clinical use of nivolumab plus ipilimumab. Univariate and multivariate logistic regression analyses showed that none of the three treatment regimens significantly affected the occurrence of cardiac adverse events. Notably, we observed that the durvalumab plus tremelimumab regimen had the highest proportion of fatal outcomes, suggesting that this combination should be used with caution in patients with potential cardiovascular risks. Given the currently limited sample sizes for different dual ICI strategies, these findings require further validation.

Cardiac adverse events often present with nonspecific symptoms, and understanding their common accompanying manifestations may aid in early detection. Our study showed that 81.4% of cases were accompanied by other adverse events, with dyspnoea, fever, and diarrhoea being among the more commonly observed clinical manifestations. A previous study demonstrated that in patients diagnosed with ICI-related myocarditis, the initial cardiac manifestations were atypical; none presented with chest pain, but rather with symptoms such as dyspnoea, fatigue, or shock.^32^ This suggests that in patients receiving ICI therapy, the emergence of such symptoms should prompt careful evaluation for potential cardiac involvement.

A recent French cohort study by Salem *et al*. reported that dual ICI represents a significant risk factor for ICI-related myotoxicity, with myocarditis frequently overlapping with myositis and/or myasthenia gravis, clinical manifestations that are associated with poorer outcomes.^33^ In our analysis, among patients treated with dual ICI therapy, myocarditis accounted for approximately 24.8% of all reported cardiac adverse events. About 28% of myocarditis cases were accompanied by myositis or myasthenia gravis, a proportion lower than the 43% reported in a recent study.^34^ The lower proportion observed in our analysis may reflect underreporting in spontaneous databases and limited clinical recognition of such complex presentations during the early years of ICI use.

This study has several limitations. As a spontaneous reporting database, it is subject to inherent selection biases, such as differences in reporting countries, geographic regions, and awareness of immune-related cardiac toxicities. With growing recognition of ICI-associated cardiotoxicity, heightened clinical awareness and improved diagnostic sensitivity have made clinicians and the public more likely to identify and report such events, which may partly explain the observed increase in the proportion of cardiac adverse events among all reported adverse events. Second, the reporting of a cardiac adverse event in FAERS does not necessarily imply a confirmed causal relationship with ICI therapy. Some cardiac events may arise from non–immune-mediated mechanisms, comorbid cardiovascular conditions, or concurrent medications. Nevertheless, inclusion of all cardiac-related PTs enabled a comprehensive assessment of the overall cardiac safety profile of ICIs. Third, the FAERS database does not contain data on the total number of patients receiving the drugs, making it impossible to calculate the incidence of adverse events. Despite these inherent limitations, the present study still provides an overview and valuable insights into cardiac adverse events associated with dual ICIs.

## Conclusion

Dual ICIs have markedly improved the efficacy of antitumour therapy and emerged as a viable treatment option across diverse cancer types. FAERS database, our study conducted a comprehensive retrospective analysis of Dual ICI therapy. We explored its association with cardiotoxicity, identifying susceptible patient subgroups and prevalent concomitant symptoms. These findings hold promise for informing clinical practice, enhancing pharmacovigilance, refining management strategies, and guiding future research endeavours.

## Supplementary Material

pvag006_Supplementary_Data

## Data Availability

The FAERS database is publicly accessible at https://www.fda.gov/drugs/surveillance/fdas-adverse-event-reporting-system-faers
