# Supplementary material for: Cardiac adverse events associated with dual immune checkpoint inhibitors: a pharmacovigilance analysis from the FDA adverse event reporting system
Source: Eur Heart J Cardiovasc Pharmacother. 2026 Jan 22;12(2):108–17. doi: 10.1093/ehjcvp/pvag006 (PMC12946970; doi:10.1093/ehjcvp/pvag006)
Supplement: pvag006_Supplementary_Data [file pvag006_supplementary_data.docx]

Supplementary Material

**Supplementary Table S1:**

**ICI-associated cardiac adverse**

| Cardiac Adverse | Cardiac Adverse | Cardiac Adverse | Cardiac Adverse |
| --- | --- | --- | --- |
| Myocardial infarction | Cardiomyopathy | Sinus tachycardia | Atrioventricular block complete |
| Atrioventricular block | Myocarditis | Atrial fibrillation | Atrial flutter |
| Arrhythmia | Pericardial effusion | Sinus bradycardia | Cardiac failure congestive |
| Angina pectoris | Pericarditis | Electrocardiogram abnormal | Coronary artery disease |
| Supraventricular tachycardia | Palpitations | Acute myocardial infarction | Tachycardia |
| Bradycardia | Cardiac failure | Endocarditis | Myocardial necrosis marker increased |
| Ventricular arrhythmia | Cardiogenic shock | Coronary artery insufficiency | Ejection fraction decreased |
| Stress cardiomyopathy | Cardiac arrest | Autoimmune myocarditis | Cardiac tamponade |
| Conduction disorder | Acute coronary syndrome | Cardiotoxicity | Prinzmetal angina |
| Cardiac asthma | Cardiac disorder | Bundle branch block left | Electrocardiogram qt prolonged |
| Atrial enlargement | Troponin increased | Angina unstable | Pericardial effusion malignant |
| Atrioventricular block second degree | Ischaemic cardiomyopathy | Myocardial ischaemia | Cardiomegaly |
| Troponin i increased | Troponin t increased | Coronary artery occlusion | Left ventricular failure |
| Bundle branch block right | Supraventricular extrasystoles | Left ventricular dysfunction | Cardiac flutter |
| Extrasystoles | Pericardial disease | Immune-mediated myocarditis | Myocardial fibrosis |
| Trifascicular block | Acute left ventricular failure | Pleuropericarditis | Pericardial haemorrhage |
| Carditis | Mitral valve disease | Tricuspid valve disease | Diastolic dysfunction |
| Ventricular tachycardia | Cardio-respiratory arrest | Electrocardiogram qrs complex prolonged | Heart alternation |
| Cardiovascular disorder | Ventricular extrasystoles | Cardiopulmonary failure | Autoimmune pericarditis |
| Cardiovascular insufficiency | Cardiovascular deconditioning | Electrocardiogram change | Ventricular dysfunction |
| Sinus node dysfunction | Cardiac failure acute | Nodal rhythm | Aortic valve stenosis |
| Cardio-respiratory distress | Cardiac dysfunction | Ventricular fibrillation | Nodal arrhythmia |
| Coronary artery stenosis | Wellens' syndrome | Bundle branch block | Pulseless electrical activity |
| Cardiac failure chronic | Pericarditis constrictive | Atrial tachycardia | Electrocardiogram q wave abnormal |
| Tachycardia induced cardiomyopathy | Subendocardial ischaemia | Cardiac valve disease | Cardiac sarcoidosis |
| Torsade de pointes | Immune-mediated pericarditis | Kounis syndrome | Atrial thrombosis |
| Aortic valve disease | Arteriosclerosis coronary artery | Aortic valve incompetence | Myopericarditis |
| Pericarditis malignant | Cardiac hypertrophy | Electrocardiogram st segment elevation | Electrocardiogram st segment depression |
| Electrocardiogram t wave abnormal | Myocardial necrosis | Ejection fraction abnormal | Pulmonary valve incompetence |
| Ventricular tachyarrhythmia | Acute right ventricular failure | Right ventricular failure | Arteriospasm coronary |
| Cardiorenal syndrome | Bradyarrhythmia | Cardiac ventricular thrombosis | Heart valve incompetence |
| Dilated cardiomyopathy | Ventricular hypokinesia | Tachyarrhythmia | Mitral valve incompetence |
| Silent myocardial infarction | Low cardiac output syndrome | Left ventricular hypertrophy | Myocardial injury |
| Arrhythmia supraventricular | Troponin abnormal | Paroxysmal atrioventricular block | Toxic cardiomyopathy |

**Supplementary Table S2:**

**Two-by-two contingency table for disproportionality analyses**

|  | Target AEs | Other AEs | Total |
| --- | --- | --- | --- |
| Target drugs | a | b | a+b |
| Other drugs | c | d | c+d |
| Total | a+c | b+d | a+b+c+d |

Abbreviation: AEs, adverse events; a, number of reports containing both the target drug and target adverse drug reaction; b, number of reports containing other adverse drug reaction of the target drug; c, number of reports containing the target adverse drug reaction of other drugs; d, number of reports containing other drugs and other adverse drug reactions.

**Supplementary Table S3:**

**Four major algorithms used for signal detection.**

| Algorithms | Equation | Criteria |
| --- | --- | --- |
| ROR | ROR=ad/b/c | lower limit of 95% CI>1, N≥3 |
|  | 95%CI=e^ln(ROR)±1.96(1/a+1/b+1/c+1/d)^0.5^ |  |
| PRR | PRR=a(c+d)/c/(a+b) | PRR≥2, χ^2^≥4, N≥3 |
|  | χ^2^=[(ad-bc)^2](a+b+c+d)/[(a+b)(c+d)(a+c)(b+d)] |  |
| BCPNN | IC=log_2_a(a+b+c+d)(a+c)(a+b) | IC025>0 |
|  | 95%CI= E(IC) ± 2V(IC)^0.5 |  |
| MGPS | EBGM=a(a+b+c+d)/(a+c)/(a+b) | EBGM05>2 |
|  | 95%CI=e^ln(EBGM)±1.96(1/a+1/b+1/c+1/d)^0.5^ |  |

Abbreviation: a, number of reports containing both the target drug and target adverse drug reaction; b, number of reports containing other adverse drug reaction of the target drug; c, number of reports containing the target adverse drug reaction of other drugs; d, number of reports containing other drugs and other adverse drug reactions. 95%CI, 95% confidence interval; N, the number of reports; χ2, chi-squared; IC, information component; IC025, the lower limit of 95% CI of the IC; E(IC), the IC expectations; V(IC), the variance of IC; EBGM, empirical Bayesian geometric mean; EBGM05, the lower limit of 95% CI of EBGM.

**Supplementary Table S4:**

**Common cardiac immune-related adverse events associated with dual ICIs and their mortality rates**

| Cardiac adverse | Numbers | Mortality (%) |
| --- | --- | --- |
| Myocarditis | 394 | 38.3248731 |
| Immune-mediated myocarditis | 213 | 28.16901408 |
| Atrial fibrillation | 194 | 20.6185567 |
| Cardiac failure | 167 | 32.33532934 |
| Pericardial effusion | 117 | 15.38461538 |
| Myocardial infarction | 88 | 42.04545455 |
| Cardiac arrest | 77 | 76.62337662 |
| Tachycardia | 73 | 19.17808219 |
| Troponin increased | 53 | 13.20754717 |
| Acute myocardial infarction | 47 | 42.55319149 |
| Sinus tachycardia | 47 | 14.89361702 |
| Cardiac disorder | 45 | 28.88888889 |
| Arrhythmia | 44 | 45.45454545 |
| Atrioventricular block complete | 44 | 40.90909091 |
| Cardiac tamponade | 44 | 15.90909091 |
| Cardiomyopathy | 44 | 31.81818182 |
| Cardio-respiratory arrest | 40 | 75 |
| Cardiogenic shock | 40 | 52.5 |
| Pericarditis | 40 | 7.5 |
| Cardiac failure congestive | 31 | 35.48387097 |
| Atrial flutter | 25 | 12 |
| Atrioventricular block | 25 | 48 |
| Palpitations | 25 | 4 |
| Ventricular tachycardia | 23 | 39.13043478 |
| Supraventricular tachycardia | 22 | 13.63636364 |
| Troponin i increased | 22 | 31.81818182 |
| Stress cardiomyopathy | 21 | 28.57142857 |

**Supplementary Table S5:**

**Signal strength of Dual ICIs Cardiac adverse events in the FAERS database**

| PT | Numbers | ROR(95%Cl) | PRR(X²) | EBGM(EBGM05) | IC(IC025) |
| --- | --- | --- | --- | --- | --- |
| Myocarditis | 394 | 30.16 ( 27.24 - 33.39 ) | 30 ( 10471.04 ) | 28.49 ( 26.16 ) | 4.83 ( 4.68 ) |
| Immune-mediated myocarditis | 213 | 151.4 ( 130.05 - 176.24 ) | 150.96 ( 24841.29 ) | 118.4 ( 104.26 ) | 6.89 ( 6.67 ) |
| Atrial fibrillation | 194 | 1.74 ( 1.51 - 2.01 ) | 1.74 ( 61.21 ) | 1.74 ( 1.55 ) | 0.8 ( 0.59 ) |
| Cardiac failure | 167 | 1.83 ( 1.57 - 2.13 ) | 1.82 ( 62.01 ) | 1.82 ( 1.6 ) | 0.86 ( 0.64 ) |
| Pericardial effusion | 117 | 4.66 ( 3.88 - 5.59 ) | 4.65 ( 332.88 ) | 4.62 ( 3.97 ) | 2.21 ( 1.94 ) |
| Myocardial infarction | 88 | 0.6 ( 0.49 - 0.75 ) | 0.61 ( 22.66 ) | 0.61 ( 0.51 ) | -0.72 ( -1.03 ) |
| Cardiac arrest | 77 | 0.96 ( 0.77 - 1.21 ) | 0.96 ( 0.1 ) | 0.96 ( 0.8 ) | -0.05 ( -0.38 ) |
| Tachycardia | 73 | 0.75 ( 0.6 - 0.95 ) | 0.75 ( 5.83 ) | 0.75 ( 0.62 ) | -0.41 ( -0.74 ) |
| Troponin increased | 53 | 6.62 ( 5.05 - 8.68 ) | 6.62 ( 249.61 ) | 6.55 ( 5.22 ) | 2.71 ( 2.32 ) |
| Sinus tachycardia | 47 | 3.47 ( 2.6 - 4.62 ) | 3.47 ( 82.02 ) | 3.45 ( 2.72 ) | 1.79 ( 1.37 ) |
| Acute myocardial infarction | 47 | 1.58 ( 1.19 - 2.11 ) | 1.58 ( 10.07 ) | 1.58 ( 1.24 ) | 0.66 ( 0.24 ) |
| Cardiac disorder | 45 | 0.45 ( 0.34 - 0.61 ) | 0.45 ( 29.52 ) | 0.45 ( 0.36 ) | -1.14 ( -1.56 ) |
| Cardiomyopathy | 44 | 3.09 ( 2.3 - 4.16 ) | 3.09 ( 61.79 ) | 3.08 ( 2.4 ) | 1.62 ( 1.19 ) |
| Atrioventricular block complete | 44 | 6.67 ( 4.96 - 8.99 ) | 6.67 ( 209.58 ) | 6.6 ( 5.15 ) | 2.72 ( 2.29 ) |
| Arrhythmia | 44 | 0.89 ( 0.66 - 1.2 ) | 0.89 ( 0.57 ) | 0.89 ( 0.7 ) | -0.16 ( -0.59 ) |
| Cardiac tamponade | 44 | 8.29 ( 6.15 - 11.16 ) | 8.28 ( 277.49 ) | 8.17 ( 6.37 ) | 3.03 ( 2.6 ) |
| Pericarditis | 40 | 1.96 ( 1.43 - 2.67 ) | 1.96 ( 18.63 ) | 1.95 ( 1.51 ) | 0.97 ( 0.51 ) |
| Cardiogenic shock | 40 | 2.51 ( 1.84 - 3.43 ) | 2.51 ( 36.18 ) | 2.5 ( 1.93 ) | 1.32 ( 0.87 ) |
| Cardio-respiratory arrest | 40 | 0.99 ( 0.73 - 1.35 ) | 0.99 ( 0 ) | 0.99 ( 0.76 ) | -0.02 ( -0.47 ) |
| Cardiac failure congestive | 31 | 0.41 ( 0.29 - 0.59 ) | 0.41 ( 25.72 ) | 0.41 ( 0.31 ) | -1.27 ( -1.78 ) |
| Atrioventricular block | 25 | 3.11 ( 2.1 - 4.6 ) | 3.11 ( 35.52 ) | 3.09 ( 2.23 ) | 1.63 ( 1.06 ) |
| Atrial flutter | 25 | 2.94 ( 1.99 - 4.36 ) | 2.94 ( 31.86 ) | 2.93 ( 2.11 ) | 1.55 ( 0.98 ) |
| Palpitations | 25 | 0.19 ( 0.13 - 0.28 ) | 0.19 ( 86.21 ) | 0.19 ( 0.14 ) | -2.39 ( -2.96 ) |
| Ventricular tachycardia | 23 | 1.45 ( 0.96 - 2.19 ) | 1.45 ( 3.23 ) | 1.45 ( 1.03 ) | 0.54 ( -0.05 ) |
| Supraventricular tachycardia | 22 | 2.37 ( 1.56 - 3.61 ) | 2.37 ( 17.39 ) | 2.37 ( 1.67 ) | 1.24 ( 0.64 ) |
| Troponin i increased | 22 | 12.77 ( 8.37 - 19.49 ) | 12.77 ( 233.12 ) | 12.5 ( 8.77 ) | 3.64 ( 3.03 ) |
| Stress cardiomyopathy | 21 | 2.89 ( 1.88 - 4.43 ) | 2.89 ( 25.73 ) | 2.88 ( 2.01 ) | 1.52 ( 0.91 ) |
| Angina pectoris | 20 | 0.71 ( 0.46 - 1.11 ) | 0.71 ( 2.28 ) | 0.72 ( 0.5 ) | -0.48 ( -1.11 ) |
| Cardiac failure acute | 20 | 2.46 ( 1.59 - 3.82 ) | 2.46 ( 17.29 ) | 2.46 ( 1.7 ) | 1.3 ( 0.66 ) |
| Bradycardia | 19 | 0.32 ( 0.2 - 0.5 ) | 0.32 ( 27.3 ) | 0.32 ( 0.22 ) | -1.64 ( -2.28 ) |
| Autoimmune myocarditis | 16 | 50.35 ( 30.17 - 84.04 ) | 50.34 ( 708.27 ) | 46.16 ( 30.07 ) | 5.53 ( 4.8 ) |
| Cardiac dysfunction | 16 | 2.96 ( 1.81 - 4.83 ) | 2.96 ( 20.59 ) | 2.94 ( 1.95 ) | 1.56 ( 0.86 ) |
| Electrocardiogram qt prolonged | 15 | 0.34 ( 0.21 - 0.57 ) | 0.34 ( 18.7 ) | 0.34 ( 0.23 ) | -1.54 ( -2.26 ) |
| Ejection fraction decreased | 14 | 0.77 ( 0.46 - 1.3 ) | 0.77 ( 0.96 ) | 0.77 ( 0.5 ) | -0.38 ( -1.12 ) |
| Cardiotoxicity | 14 | 1.23 ( 0.73 - 2.08 ) | 1.23 ( 0.6 ) | 1.23 ( 0.79 ) | 0.3 ( -0.45 ) |
| Coronary artery disease | 12 | 0.55 ( 0.31 - 0.98 ) | 0.55 ( 4.29 ) | 0.55 ( 0.35 ) | -0.85 ( -1.65 ) |
| Troponin t increased | 11 | 8.05 ( 4.44 - 14.6 ) | 8.05 ( 66.9 ) | 7.94 ( 4.83 ) | 2.99 ( 2.15 ) |
| Ventricular fibrillation | 11 | 1.09 ( 0.6 - 1.96 ) | 1.09 ( 0.07 ) | 1.08 ( 0.66 ) | 0.12 ( -0.72 ) |
| Myocardial necrosis marker increased | 10 | 4.34 ( 2.33 - 8.08 ) | 4.34 ( 25.47 ) | 4.31 ( 2.56 ) | 2.11 ( 1.23 ) |
| Acute coronary syndrome | 10 | 1.2 ( 0.64 - 2.23 ) | 1.2 ( 0.32 ) | 1.2 ( 0.71 ) | 0.26 ( -0.61 ) |
| Cardiovascular disorder | 10 | 0.63 ( 0.34 - 1.17 ) | 0.63 ( 2.17 ) | 0.63 ( 0.38 ) | -0.67 ( -1.54 ) |
| Coronary artery stenosis | 9 | 2.43 ( 1.26 - 4.67 ) | 2.43 ( 7.51 ) | 2.42 ( 1.4 ) | 1.28 ( 0.36 ) |
| Sinus bradycardia | 8 | 0.81 ( 0.4 - 1.62 ) | 0.81 ( 0.36 ) | 0.81 ( 0.45 ) | -0.31 ( -1.27 ) |
| Bundle branch block right | 8 | 2.06 ( 1.03 - 4.13 ) | 2.06 ( 4.36 ) | 2.06 ( 1.15 ) | 1.04 ( 0.08 ) |
| Endocarditis | 7 | 1.22 ( 0.58 - 2.56 ) | 1.22 ( 0.27 ) | 1.22 ( 0.65 ) | 0.28 ( -0.74 ) |
| Ventricular arrhythmia | 7 | 1.66 ( 0.79 - 3.48 ) | 1.66 ( 1.82 ) | 1.66 ( 0.89 ) | 0.73 ( -0.29 ) |
| Ventricular extrasystoles | 7 | 0.76 ( 0.36 - 1.6 ) | 0.76 ( 0.51 ) | 0.76 ( 0.41 ) | -0.39 ( -1.41 ) |
| Atrioventricular block second degree | 6 | 2.05 ( 0.92 - 4.56 ) | 2.04 ( 3.19 ) | 2.04 ( 1.04 ) | 1.03 ( -0.06 ) |
| Myocardial ischaemia | 6 | 0.67 ( 0.3 - 1.5 ) | 0.67 ( 0.96 ) | 0.67 ( 0.34 ) | -0.57 ( -1.67 ) |
| Left ventricular dysfunction | 6 | 0.81 ( 0.36 - 1.8 ) | 0.81 ( 0.27 ) | 0.81 ( 0.41 ) | -0.3 ( -1.4 ) |
| Ischaemic cardiomyopathy | 5 | 2.36 ( 0.98 - 5.69 ) | 2.36 ( 3.91 ) | 2.36 ( 1.13 ) | 1.24 ( 0.06 ) |
| Cardiomegaly | 5 | 0.5 ( 0.21 - 1.21 ) | 0.5 ( 2.48 ) | 0.5 ( 0.24 ) | -0.99 ( -2.17 ) |
| Diastolic dysfunction | 5 | 1.64 ( 0.68 - 3.95 ) | 1.64 ( 1.25 ) | 1.64 ( 0.79 ) | 0.71 ( -0.47 ) |
| Sinus node dysfunction | 5 | 1.76 ( 0.73 - 4.23 ) | 1.76 ( 1.63 ) | 1.76 ( 0.84 ) | 0.81 ( -0.37 ) |
| Bundle branch block left | 4 | 1.02 ( 0.38 - 2.72 ) | 1.02 ( 0 ) | 1.02 ( 0.45 ) | 0.03 ( -1.26 ) |
| Coronary artery occlusion | 4 | 0.36 ( 0.13 - 0.95 ) | 0.36 ( 4.65 ) | 0.36 ( 0.16 ) | -1.49 ( -2.78 ) |
| Left ventricular failure | 4 | 1.09 ( 0.41 - 2.91 ) | 1.09 ( 0.03 ) | 1.09 ( 0.48 ) | 0.12 ( -1.17 ) |
| Pleuropericarditis | 4 | 9.94 ( 3.7 - 26.73 ) | 9.94 ( 31.59 ) | 9.78 ( 4.28 ) | 3.29 ( 1.99 ) |
| Carditis | 4 | 8.74 ( 3.26 - 23.48 ) | 8.74 ( 27 ) | 8.62 ( 3.77 ) | 3.11 ( 1.8 ) |
| Ventricular dysfunction | 4 | 2.41 ( 0.9 - 6.45 ) | 2.41 ( 3.3 ) | 2.41 ( 1.06 ) | 1.27 ( -0.03 ) |
| Immune-mediated pericarditis | 4 | 70.24 ( 24.79 - 198.98 ) | 70.23 ( 241.79 ) | 62.32 ( 26.08 ) | 5.96 ( 4.58 ) |
| Pericarditis malignant | 4 | 26.55 ( 9.73 - 72.44 ) | 26.55 ( 93.78 ) | 25.36 ( 10.95 ) | 4.66 ( 3.34 ) |
| Mitral valve incompetence | 4 | 0.44 ( 0.16 - 1.17 ) | 0.44 ( 2.86 ) | 0.44 ( 0.19 ) | -1.19 ( -2.48 ) |
| Electrocardiogram abnormal | 3 | 0.38 ( 0.12 - 1.18 ) | 0.38 ( 3.02 ) | 0.38 ( 0.15 ) | -1.39 ( -2.83 ) |
| Angina unstable | 3 | 0.57 ( 0.18 - 1.77 ) | 0.57 ( 0.97 ) | 0.57 ( 0.22 ) | -0.81 ( -2.25 ) |
| Electrocardiogram qrs complex prolonged | 3 | 0.52 ( 0.17 - 1.62 ) | 0.52 ( 1.31 ) | 0.52 ( 0.2 ) | -0.93 ( -2.38 ) |
| Autoimmune pericarditis | 3 | 163.3 ( 44.94 - 593.38 ) | 163.29 ( 372.23 ) | 125.84 ( 42.75 ) | 6.98 ( 5.34 ) |
| Pulseless electrical activity | 3 | 0.58 ( 0.19 - 1.79 ) | 0.58 ( 0.93 ) | 0.58 ( 0.22 ) | -0.79 ( -2.23 ) |
| Cardiac failure chronic | 3 | 0.51 ( 0.17 - 1.59 ) | 0.51 ( 1.39 ) | 0.51 ( 0.2 ) | -0.96 ( -2.41 ) |
| Cardiac sarcoidosis | 3 | 11.58 ( 3.69 - 36.34 ) | 11.58 ( 28.4 ) | 11.36 ( 4.36 ) | 3.51 ( 2.04 ) |
| Torsade de pointes | 3 | 0.38 ( 0.12 - 1.19 ) | 0.38 ( 2.99 ) | 0.38 ( 0.15 ) | -1.38 ( -2.83 ) |
| Atrial thrombosis | 3 | 1.19 ( 0.38 - 3.69 ) | 1.19 ( 0.09 ) | 1.19 ( 0.46 ) | 0.25 ( -1.2 ) |
| Arteriosclerosis coronary artery | 3 | 0.53 ( 0.17 - 1.66 ) | 0.53 ( 1.21 ) | 0.54 ( 0.21 ) | -0.9 ( -2.35 ) |
| Aortic valve incompetence | 3 | 0.9 ( 0.29 - 2.79 ) | 0.9 ( 0.03 ) | 0.9 ( 0.35 ) | -0.15 ( -1.6 ) |
| Myopericarditis | 3 | 4.52 ( 1.45 - 14.09 ) | 4.52 ( 8.17 ) | 4.49 ( 1.74 ) | 2.17 ( 0.72 ) |
| Electrocardiogram st segment elevation | 3 | 1.01 ( 0.33 - 3.15 ) | 1.01 ( 0 ) | 1.01 ( 0.39 ) | 0.02 ( -1.42 ) |
| Ventricular hypokinesia | 3 | 0.94 ( 0.3 - 2.91 ) | 0.94 ( 0.01 ) | 0.94 ( 0.36 ) | -0.09 ( -1.54 ) |
| Conduction disorder | 2 | 1.27 ( 0.32 - 5.11 ) | 1.27 ( 0.12 ) | 1.27 ( 0.4 ) | 0.35 ( -1.32 ) |
| Prinzmetal angina | 2 | 1.79 ( 0.45 - 7.18 ) | 1.79 ( 0.7 ) | 1.79 ( 0.56 ) | 0.84 ( -0.83 ) |
| Pericardial effusion malignant | 2 | 10.89 ( 2.69 - 44.14 ) | 10.89 ( 17.6 ) | 10.69 ( 3.31 ) | 3.42 ( 1.73 ) |
| Cardiac flutter | 2 | 0.27 ( 0.07 - 1.09 ) | 0.27 ( 3.88 ) | 0.27 ( 0.09 ) | -1.87 ( -3.54 ) |
| Pericardial disease | 2 | 6.44 ( 1.6 - 25.97 ) | 6.44 ( 9.09 ) | 6.38 ( 1.99 ) | 2.67 ( 0.99 ) |
| Trifascicular block | 2 | 14.52 ( 3.56 - 59.12 ) | 14.51 ( 24.51 ) | 14.16 ( 4.37 ) | 3.82 ( 2.13 ) |
| Cardiopulmonary failure | 2 | 0.55 ( 0.14 - 2.21 ) | 0.55 ( 0.72 ) | 0.55 ( 0.17 ) | -0.85 ( -2.52 ) |
| Cardiovascular insufficiency | 2 | 1.12 ( 0.28 - 4.49 ) | 1.12 ( 0.03 ) | 1.12 ( 0.35 ) | 0.16 ( -1.5 ) |
| Electrocardiogram change | 2 | 3.31 ( 0.82 - 13.29 ) | 3.31 ( 3.2 ) | 3.29 ( 1.03 ) | 1.72 ( 0.05 ) |
| Pericarditis constrictive | 2 | 4.43 ( 1.1 - 17.8 ) | 4.43 ( 5.26 ) | 4.4 ( 1.37 ) | 2.14 ( 0.46 ) |
| Atrial tachycardia | 2 | 0.87 ( 0.22 - 3.47 ) | 0.87 ( 0.04 ) | 0.87 ( 0.27 ) | -0.21 ( -1.87 ) |
| Cardiac valve disease | 2 | 0.35 ( 0.09 - 1.4 ) | 0.35 ( 2.4 ) | 0.35 ( 0.11 ) | -1.51 ( -3.17 ) |
| Cardiac hypertrophy | 2 | 1.94 ( 0.48 - 7.79 ) | 1.94 ( 0.91 ) | 1.94 ( 0.61 ) | 0.96 ( -0.71 ) |
| Electrocardiogram st segment depression | 2 | 1.09 ( 0.27 - 4.36 ) | 1.09 ( 0.01 ) | 1.09 ( 0.34 ) | 0.12 ( -1.54 ) |
| Dilated cardiomyopathy | 2 | 2.05 ( 0.51 - 8.22 ) | 2.05 ( 1.07 ) | 2.05 ( 0.64 ) | 1.03 ( -0.64 ) |
| Left ventricular hypertrophy | 2 | 0.5 ( 0.13 - 2.02 ) | 0.5 ( 0.98 ) | 0.5 ( 0.16 ) | -0.99 ( -2.65 ) |
| Coronary artery insufficiency | 1 | 4.16 ( 0.58 - 29.72 ) | 4.16 ( 2.38 ) | 4.13 ( 0.8 ) | 2.05 ( -0.01 ) |
| Cardiac asthma | 1 | 7.07 ( 0.98 - 50.83 ) | 7.07 ( 5.14 ) | 6.99 ( 1.34 ) | 2.81 ( 0.74 ) |
| Atrial enlargement | 1 | 2.37 ( 0.33 - 16.87 ) | 2.37 ( 0.79 ) | 2.36 ( 0.46 ) | 1.24 ( -0.81 ) |
| Supraventricular extrasystoles | 1 | 0.35 ( 0.05 - 2.46 ) | 0.35 ( 1.23 ) | 0.35 ( 0.07 ) | -1.53 ( -3.57 ) |
| Extrasystoles | 1 | 0.15 ( 0.02 - 1.03 ) | 0.15 ( 5.02 ) | 0.15 ( 0.03 ) | -2.78 ( -4.82 ) |
| Myocardial fibrosis | 1 | 1.11 ( 0.16 - 7.87 ) | 1.11 ( 0.01 ) | 1.11 ( 0.21 ) | 0.15 ( -1.9 ) |
| Acute left ventricular failure | 1 | 1.12 ( 0.16 - 8 ) | 1.12 ( 0.01 ) | 1.12 ( 0.22 ) | 0.17 ( -1.88 ) |
| Pericardial haemorrhage | 1 | 0.38 ( 0.05 - 2.71 ) | 0.38 ( 1 ) | 0.38 ( 0.07 ) | -1.39 ( -3.43 ) |
| Mitral valve disease | 1 | 0.63 ( 0.09 - 4.45 ) | 0.63 ( 0.22 ) | 0.63 ( 0.12 ) | -0.67 ( -2.72 ) |
| Tricuspid valve disease | 1 | 2.52 ( 0.35 - 17.97 ) | 2.52 ( 0.91 ) | 2.51 ( 0.49 ) | 1.33 ( -0.72 ) |
| Heart alternation | 1 | 9.9 ( 1.37 - 71.52 ) | 9.9 ( 7.85 ) | 9.74 ( 1.86 ) | 3.28 ( 1.21 ) |
| Cardiovascular deconditioning | 1 | 28.65 ( 3.83 - 214.01 ) | 28.65 ( 25.35 ) | 27.27 ( 5.07 ) | 4.77 ( 2.63 ) |
| Nodal rhythm | 1 | 0.88 ( 0.12 - 6.23 ) | 0.88 ( 0.02 ) | 0.88 ( 0.17 ) | -0.19 ( -2.23 ) |
| Aortic valve stenosis | 1 | 0.53 ( 0.07 - 3.73 ) | 0.53 ( 0.43 ) | 0.53 ( 0.1 ) | -0.93 ( -2.97 ) |
| Cardio-respiratory distress | 1 | 3.73 ( 0.52 - 26.65 ) | 3.73 ( 1.98 ) | 3.71 ( 0.72 ) | 1.89 ( -0.16 ) |
| Nodal arrhythmia | 1 | 1.56 ( 0.22 - 11.1 ) | 1.56 ( 0.2 ) | 1.56 ( 0.3 ) | 0.64 ( -1.41 ) |
| Wellens' syndrome | 1 | 20.16 ( 2.74 - 148.36 ) | 20.16 ( 17.56 ) | 19.48 ( 3.67 ) | 4.28 ( 2.17 ) |
| Bundle branch block | 1 | 1.24 ( 0.17 - 8.8 ) | 1.24 ( 0.05 ) | 1.24 ( 0.24 ) | 0.31 ( -1.74 ) |
| Electrocardiogram q wave abnormal | 1 | 4.77 ( 0.67 - 34.19 ) | 4.77 ( 2.96 ) | 4.74 ( 0.91 ) | 2.25 ( 0.19 ) |
| Tachycardia induced cardiomyopathy | 1 | 5.04 ( 0.7 - 36.11 ) | 5.04 ( 3.21 ) | 5 ( 0.96 ) | 2.32 ( 0.26 ) |
| Subendocardial ischaemia | 1 | 11.58 ( 1.6 - 83.94 ) | 11.58 ( 9.47 ) | 11.36 ( 2.17 ) | 3.51 ( 1.42 ) |
| Kounis syndrome | 1 | 0.33 ( 0.05 - 2.37 ) | 0.33 ( 1.33 ) | 0.33 ( 0.06 ) | -1.58 ( -3.62 ) |
| Aortic valve disease | 1 | 0.61 ( 0.09 - 4.33 ) | 0.61 ( 0.25 ) | 0.61 ( 0.12 ) | -0.71 ( -2.76 ) |
| Electrocardiogram t wave abnormal | 1 | 0.81 ( 0.11 - 5.78 ) | 0.81 ( 0.04 ) | 0.81 ( 0.16 ) | -0.3 ( -2.34 ) |
| Myocardial necrosis | 1 | 2.79 ( 0.39 - 19.92 ) | 2.79 ( 1.14 ) | 2.78 ( 0.54 ) | 1.48 ( -0.57 ) |
| Ejection fraction abnormal | 1 | 0.57 ( 0.08 - 4.02 ) | 0.57 ( 0.33 ) | 0.57 ( 0.11 ) | -0.82 ( -2.86 ) |
| Pulmonary valve incompetence | 1 | 0.91 ( 0.13 - 6.45 ) | 0.91 ( 0.01 ) | 0.91 ( 0.18 ) | -0.14 ( -2.18 ) |
| Ventricular tachyarrhythmia | 1 | 2.42 ( 0.34 - 17.25 ) | 2.42 ( 0.83 ) | 2.41 ( 0.47 ) | 1.27 ( -0.78 ) |
| Acute right ventricular failure | 1 | 4.46 ( 0.62 - 31.93 ) | 4.46 ( 2.66 ) | 4.43 ( 0.85 ) | 2.15 ( 0.09 ) |
| Right ventricular failure | 1 | 0.12 ( 0.02 - 0.86 ) | 0.12 ( 6.34 ) | 0.12 ( 0.02 ) | -3.04 ( -5.08 ) |
| Arteriospasm coronary | 1 | 0.24 ( 0.03 - 1.73 ) | 0.24 ( 2.34 ) | 0.24 ( 0.05 ) | -2.03 ( -4.08 ) |
| Cardiorenal syndrome | 1 | 1.19 ( 0.17 - 8.44 ) | 1.19 ( 0.03 ) | 1.19 ( 0.23 ) | 0.25 ( -1.8 ) |
| Bradyarrhythmia | 1 | 0.64 ( 0.09 - 4.56 ) | 0.64 ( 0.2 ) | 0.64 ( 0.12 ) | -0.64 ( -2.68 ) |
| Cardiac ventricular thrombosis | 1 | 0.64 ( 0.09 - 4.55 ) | 0.64 ( 0.2 ) | 0.64 ( 0.12 ) | -0.64 ( -2.69 ) |
| Heart valve incompetence | 1 | 0.3 ( 0.04 - 2.16 ) | 0.3 ( 1.59 ) | 0.3 ( 0.06 ) | -1.71 ( -3.76 ) |
| Tachyarrhythmia | 1 | 0.4 ( 0.06 - 2.85 ) | 0.4 ( 0.9 ) | 0.4 ( 0.08 ) | -1.32 ( -3.36 ) |
| Silent myocardial infarction | 1 | 2.37 ( 0.33 - 16.87 ) | 2.37 ( 0.79 ) | 2.36 ( 0.46 ) | 1.24 ( -0.81 ) |
| Low cardiac output syndrome | 1 | 2.42 ( 0.34 - 17.25 ) | 2.42 ( 0.83 ) | 2.41 ( 0.47 ) | 1.27 ( -0.78 ) |
| Myocardial injury | 1 | 0.93 ( 0.13 - 6.62 ) | 0.93 ( 0.01 ) | 0.93 ( 0.18 ) | -0.1 ( -2.15 ) |
| Arrhythmia supraventricular | 1 | 1.07 ( 0.15 - 7.58 ) | 1.07 ( 0 ) | 1.07 ( 0.21 ) | 0.09 ( -1.95 ) |
| Troponin abnormal | 1 | 6.12 ( 0.85 - 43.9 ) | 6.12 ( 4.23 ) | 6.06 ( 1.16 ) | 2.6 ( 0.54 ) |
| Paroxysmal atrioventricular block | 1 | 13.96 ( 1.92 - 101.59 ) | 13.96 ( 11.73 ) | 13.63 ( 2.59 ) | 3.77 ( 1.68 ) |
| Toxic cardiomyopathy | 1 | 3.38 ( 0.47 - 24.15 ) | 3.38 ( 1.67 ) | 3.37 ( 0.65 ) | 1.75 ( -0.3 ) |

Abbreviation: ROR, reporting odds ratio; PRR, proportional reporting ratio; EBGM, empirical Bayesian geometric mean; EBGM05, the lower limit of the 95% CI of EBGM; IC, information component; IC025, the lower limit of the 95% CI of the IC; CI, confidence interval; PT, preferred term.

**Supplementary Table S6:**

**Signal strength of pembrolizumab plus ipilimumab Cardiac adverse events in the FAERS database**

| PT | Numbers | ROR(95%Cl) | PRR(X²) | EBGM(EBGM05) | IC(IC025) |
| --- | --- | --- | --- | --- | --- |
| Immune-mediated myocarditis | 8 | 181.6 ( 90.42 - 364.72 ) | 180.81 ( 1418.86 ) | 179.34 ( 100.06 ) | 7.49 ( 6.52 ) |
| Myocarditis | 5 | 14.63 ( 6.08 - 35.19 ) | 14.59 ( 63.25 ) | 14.58 ( 6.99 ) | 3.87 ( 2.69 ) |
| Cardiomyopathy | 3 | 8.47 ( 2.73 - 26.31 ) | 8.46 ( 19.74 ) | 8.46 ( 3.28 ) | 3.08 ( 1.64 ) |
| Troponin increased | 2 | 9.98 ( 2.49 - 39.94 ) | 9.97 ( 16.13 ) | 9.96 ( 3.12 ) | 3.32 ( 1.65 ) |
| Tachycardia | 2 | 0.83 ( 0.21 - 3.34 ) | 0.83 ( 0.07 ) | 0.83 ( 0.26 ) | -0.26 ( -1.93 ) |
| Myocardial necrosis marker increased | 2 | 34.86 ( 8.7 - 139.65 ) | 34.82 ( 65.6 ) | 34.77 ( 10.89 ) | 5.12 ( 3.45 ) |
| Cardiac failure | 2 | 0.88 ( 0.22 - 3.52 ) | 0.88 ( 0.03 ) | 0.88 ( 0.28 ) | -0.19 ( -1.85 ) |
| Sinus bradycardia | 1 | 4.08 ( 0.57 - 28.99 ) | 4.08 ( 2.32 ) | 4.08 ( 0.79 ) | 2.03 ( -0.01 ) |
| Pericardial effusion | 1 | 1.59 ( 0.22 - 11.32 ) | 1.59 ( 0.22 ) | 1.59 ( 0.31 ) | 0.67 ( -1.37 ) |
| Palpitations | 1 | 0.31 ( 0.04 - 2.18 ) | 0.31 ( 1.56 ) | 0.31 ( 0.06 ) | -1.7 ( -3.74 ) |
| Myocardial infarction | 1 | 0.28 ( 0.04 - 1.97 ) | 0.28 ( 1.88 ) | 0.28 ( 0.05 ) | -1.85 ( -3.89 ) |
| Dilated cardiomyopathy | 1 | 41.36 ( 5.81 - 294.31 ) | 41.34 ( 39.29 ) | 41.26 ( 7.99 ) | 5.37 ( 3.32 ) |
| Cardiac sarcoidosis | 1 | 153.86 ( 21.51 - 1100.42 ) | 153.78 ( 150.73 ) | 152.72 ( 29.44 ) | 7.25 ( 5.2 ) |
| Cardiac failure congestive | 1 | 0.54 ( 0.08 - 3.83 ) | 0.54 ( 0.39 ) | 0.54 ( 0.1 ) | -0.89 ( -2.93 ) |
| Cardiac disorder | 1 | 0.41 ( 0.06 - 2.89 ) | 0.41 ( 0.86 ) | 0.41 ( 0.08 ) | -1.3 ( -3.34 ) |
| Atrioventricular block complete | 1 | 6.06 ( 0.85 - 43.03 ) | 6.05 ( 4.22 ) | 6.05 ( 1.17 ) | 2.6 ( 0.56 ) |
| Atrial flutter | 1 | 4.73 ( 0.67 - 33.61 ) | 4.73 ( 2.94 ) | 4.73 ( 0.92 ) | 2.24 ( 0.2 ) |
| Atrial fibrillation | 1 | 0.36 ( 0.05 - 2.57 ) | 0.36 ( 1.13 ) | 0.36 ( 0.07 ) | -1.47 ( -3.51 ) |
| Arrhythmia | 1 | 0.82 ( 0.12 - 5.81 ) | 0.82 ( 0.04 ) | 0.82 ( 0.16 ) | -0.29 ( -2.33 ) |

Abbreviation: ROR, reporting odds ratio; PRR, proportional reporting ratio; EBGM, empirical Bayesian geometric mean; EBGM05, the lower limit of the 95% CI of EBGM; IC, information component; IC025, the lower limit of the 95% CI of the IC; CI, confidence interval; PT, preferred term.

**Supplementary Table S7:**

**Signal strength of durvalumab plus tremelimumab Cardiac adverse events in the FAERS database**

| PT | Numbers | ROR(95%Cl) | PRR(X²) | EBGM(EBGM05) | IC(IC025) |
| --- | --- | --- | --- | --- | --- |
| Myocarditis | 40 | 50.48 ( 36.94 - 68.97 ) | 50.01 ( 1911.44 ) | 49.75 ( 38.31 ) | 5.64 ( 5.18 ) |
| Immune-mediated myocarditis | 14 | 136.24 ( 80.32 - 231.11 ) | 135.8 ( 1846.57 ) | 133.87 ( 86.03 ) | 7.06 ( 6.31 ) |
| Pericardial effusion | 12 | 8.18 ( 4.64 - 14.42 ) | 8.16 ( 75.38 ) | 8.16 ( 5.08 ) | 3.03 ( 2.23 ) |
| Cardiac failure | 9 | 1.69 ( 0.88 - 3.25 ) | 1.69 ( 2.53 ) | 1.69 ( 0.98 ) | 0.76 ( -0.16 ) |
| Myocardial infarction | 8 | 0.95 ( 0.47 - 1.9 ) | 0.95 ( 0.02 ) | 0.95 ( 0.53 ) | -0.08 ( -1.04 ) |
| Atrial flutter | 7 | 14.16 ( 6.74 - 29.73 ) | 14.14 ( 85.32 ) | 14.12 ( 7.59 ) | 3.82 ( 2.8 ) |
| Pericarditis | 6 | 5.05 ( 2.27 - 11.24 ) | 5.04 ( 19.43 ) | 5.04 ( 2.58 ) | 2.33 ( 1.24 ) |
| Atrial fibrillation | 6 | 0.93 ( 0.42 - 2.06 ) | 0.93 ( 0.04 ) | 0.93 ( 0.47 ) | -0.11 ( -1.2 ) |
| Cardiomyopathy | 5 | 6.02 ( 2.5 - 14.48 ) | 6.02 ( 20.91 ) | 6.01 ( 2.89 ) | 2.59 ( 1.41 ) |
| Cardiac arrest | 4 | 0.86 ( 0.32 - 2.3 ) | 0.86 ( 0.09 ) | 0.86 ( 0.38 ) | -0.21 ( -1.51 ) |
| Cardiac tamponade | 4 | 12.81 ( 4.8 - 34.16 ) | 12.8 ( 43.44 ) | 12.78 ( 5.62 ) | 3.68 ( 2.38 ) |
| Acute myocardial infarction | 4 | 2.32 ( 0.87 - 6.18 ) | 2.32 ( 2.99 ) | 2.32 ( 1.02 ) | 1.21 ( -0.08 ) |
| Troponin increased | 4 | 8.51 ( 3.19 - 22.71 ) | 8.51 ( 26.48 ) | 8.5 ( 3.74 ) | 3.09 ( 1.8 ) |
| Tachycardia | 4 | 0.71 ( 0.27 - 1.9 ) | 0.71 ( 0.47 ) | 0.71 ( 0.31 ) | -0.49 ( -1.78 ) |
| Supraventricular tachycardia | 3 | 5.56 ( 1.79 - 17.24 ) | 5.55 ( 11.2 ) | 5.55 ( 2.15 ) | 2.47 ( 1.03 ) |
| Cardiac failure acute | 3 | 6.34 ( 2.04 - 19.69 ) | 6.34 ( 13.49 ) | 6.34 ( 2.46 ) | 2.66 ( 1.22 ) |
| Cardiac failure congestive | 2 | 0.46 ( 0.11 - 1.84 ) | 0.46 ( 1.27 ) | 0.46 ( 0.14 ) | -1.12 ( -2.79 ) |
| Atrioventricular block complete | 2 | 5.17 ( 1.29 - 20.67 ) | 5.17 ( 6.71 ) | 5.16 ( 1.62 ) | 2.37 ( 0.7 ) |
| Ejection fraction decreased | 2 | 1.89 ( 0.47 - 7.58 ) | 1.89 ( 0.84 ) | 1.89 ( 0.59 ) | 0.92 ( -0.75 ) |
| Cardio-respiratory arrest | 2 | 0.85 ( 0.21 - 3.4 ) | 0.85 ( 0.05 ) | 0.85 ( 0.27 ) | -0.23 ( -1.9 ) |
| Cardiac disorder | 2 | 0.35 ( 0.09 - 1.39 ) | 0.35 ( 2.45 ) | 0.35 ( 0.11 ) | -1.52 ( -3.19 ) |
| Pericarditis malignant | 2 | 223.43 ( 54.95 - 908.46 ) | 223.33 ( 432.37 ) | 218.16 ( 67.46 ) | 7.77 ( 6.07 ) |
| Arrhythmia | 2 | 0.7 ( 0.17 - 2.79 ) | 0.7 ( 0.26 ) | 0.7 ( 0.22 ) | -0.52 ( -2.18 ) |
| Electrocardiogram abnormal | 2 | 4.38 ( 1.09 - 17.51 ) | 4.37 ( 5.2 ) | 4.37 ( 1.37 ) | 2.13 ( 0.46 ) |
| Troponin t increased | 1 | 12.44 ( 1.75 - 88.47 ) | 12.44 ( 10.51 ) | 12.42 ( 2.41 ) | 3.64 ( 1.59 ) |
| Stress cardiomyopathy | 1 | 2.36 ( 0.33 - 16.73 ) | 2.36 ( 0.78 ) | 2.36 ( 0.46 ) | 1.24 ( -0.81 ) |
| Autoimmune myocarditis | 1 | 49.9 ( 6.99 - 356.21 ) | 49.89 ( 47.66 ) | 49.63 ( 9.58 ) | 5.63 ( 3.58 ) |
| Acute coronary syndrome | 1 | 2.06 ( 0.29 - 14.62 ) | 2.06 ( 0.54 ) | 2.06 ( 0.4 ) | 1.04 ( -1 ) |
| Sinus tachycardia | 1 | 1.26 ( 0.18 - 8.97 ) | 1.26 ( 0.05 ) | 1.26 ( 0.24 ) | 0.34 ( -1.7 ) |
| Bradyarrhythmia | 1 | 11.06 ( 1.56 - 78.65 ) | 11.06 ( 9.14 ) | 11.05 ( 2.14 ) | 3.47 ( 1.42 ) |
| Ventricular hypokinesia | 1 | 5.38 ( 0.76 - 38.24 ) | 5.38 ( 3.57 ) | 5.38 ( 1.04 ) | 2.43 ( 0.39 ) |
| Myocardial necrosis marker increased | 1 | 7.42 ( 1.04 - 52.75 ) | 7.42 ( 5.55 ) | 7.42 ( 1.44 ) | 2.89 ( 0.85 ) |
| Ventricular tachycardia | 1 | 1.09 ( 0.15 - 7.71 ) | 1.09 ( 0.01 ) | 1.09 ( 0.21 ) | 0.12 ( -1.92 ) |
| Low cardiac output syndrome | 1 | 41.7 ( 5.85 - 297.38 ) | 41.69 ( 39.54 ) | 41.51 ( 8.02 ) | 5.38 ( 3.33 ) |
| Coronary artery stenosis | 1 | 4.63 ( 0.65 - 32.88 ) | 4.63 ( 2.84 ) | 4.63 ( 0.9 ) | 2.21 ( 0.17 ) |
| Paroxysmal atrioventricular block | 1 | 240.56 ( 33.04 - 1751.4 ) | 240.5 ( 232.55 ) | 234.52 ( 44.54 ) | 7.87 ( 5.78 ) |
| Atrioventricular block second degree | 1 | 5.86 ( 0.82 - 41.61 ) | 5.85 ( 4.02 ) | 5.85 ( 1.13 ) | 2.55 ( 0.51 ) |
| Myocardial ischaemia | 1 | 1.93 ( 0.27 - 13.68 ) | 1.93 ( 0.45 ) | 1.93 ( 0.37 ) | 0.95 ( -1.1 ) |

Abbreviation: ROR, reporting odds ratio; PRR, proportional reporting ratio; EBGM, empirical Bayesian geometric mean; EBGM05, the lower limit of the 95% CI of EBGM; IC, information component; IC025, the lower limit of the 95% CI of the IC; CI, confidence interval; PT, preferred term.

**Supplementary Table S8:**

**Signal strength of Nivolumab plus Ipilimumab Cardiac adverse events in the FAERS database**

| PT | Numbers | ROR(95%Cl) | PRR(X²) | EBGM(EBGM05) | IC(IC025) |
| --- | --- | --- | --- | --- | --- |
| Myocarditis | 349 | 28.93 ( 25.98 - 32.22 ) | 28.79 ( 8928.76 ) | 27.5 ( 25.13 ) | 4.78 ( 4.62 ) |
| Immune-mediated myocarditis | 191 | 143.84 ( 122.79 - 168.5 ) | 143.44 ( 21756.45 ) | 115.71 ( 101.36 ) | 6.85 ( 6.63 ) |
| Atrial fibrillation | 187 | 1.83 ( 1.59 - 2.12 ) | 1.83 ( 70.28 ) | 1.83 ( 1.62 ) | 0.87 ( 0.66 ) |
| Cardiac failure | 156 | 1.86 ( 1.59 - 2.18 ) | 1.86 ( 61.53 ) | 1.85 ( 1.63 ) | 0.89 ( 0.66 ) |
| Pericardial effusion | 104 | 4.51 ( 3.72 - 5.47 ) | 4.5 ( 281.5 ) | 4.48 ( 3.81 ) | 2.16 ( 1.88 ) |
| Myocardial infarction | 79 | 0.59 ( 0.47 - 0.74 ) | 0.59 ( 22.2 ) | 0.59 ( 0.49 ) | -0.75 ( -1.08 ) |
| Cardiac arrest | 73 | 1 ( 0.79 - 1.25 ) | 1 ( 0 ) | 1 ( 0.82 ) | -0.01 ( -0.34 ) |
| Tachycardia | 67 | 0.75 ( 0.59 - 0.96 ) | 0.75 ( 5.34 ) | 0.75 ( 0.62 ) | -0.41 ( -0.76 ) |
| Troponin increased | 47 | 6.39 ( 4.79 - 8.52 ) | 6.39 ( 211.21 ) | 6.33 ( 4.97 ) | 2.66 ( 2.24 ) |
| Sinus tachycardia | 46 | 3.7 ( 2.77 - 4.95 ) | 3.7 ( 90.02 ) | 3.68 ( 2.89 ) | 1.88 ( 1.46 ) |
| Acute myocardial infarction | 43 | 1.58 ( 1.17 - 2.13 ) | 1.58 ( 9.09 ) | 1.58 ( 1.23 ) | 0.66 ( 0.22 ) |
| Cardiac disorder | 42 | 0.46 ( 0.34 - 0.62 ) | 0.46 ( 26.31 ) | 0.46 ( 0.36 ) | -1.11 ( -1.55 ) |
| Atrioventricular block complete | 41 | 6.77 ( 4.98 - 9.22 ) | 6.77 ( 199.37 ) | 6.7 ( 5.18 ) | 2.75 ( 2.3 ) |
| Arrhythmia | 41 | 0.91 ( 0.67 - 1.23 ) | 0.91 ( 0.39 ) | 0.91 ( 0.7 ) | -0.14 ( -0.59 ) |
| Cardiogenic shock | 40 | 2.74 ( 2.01 - 3.73 ) | 2.74 ( 43.86 ) | 2.73 ( 2.1 ) | 1.45 ( 1 ) |
| Cardiac tamponade | 40 | 8.2 ( 6 - 11.2 ) | 8.19 ( 249.23 ) | 8.1 ( 6.24 ) | 3.02 ( 2.56 ) |
| Cardio-respiratory arrest | 38 | 1.02 ( 0.75 - 1.41 ) | 1.02 ( 0.02 ) | 1.02 ( 0.78 ) | 0.03 ( -0.43 ) |
| Cardiomyopathy | 36 | 2.75 ( 1.98 - 3.82 ) | 2.75 ( 39.95 ) | 2.74 ( 2.09 ) | 1.46 ( 0.98 ) |
| Pericarditis | 34 | 1.81 ( 1.29 - 2.54 ) | 1.81 ( 12.32 ) | 1.81 ( 1.36 ) | 0.86 ( 0.37 ) |
| Cardiac failure congestive | 28 | 0.41 ( 0.28 - 0.59 ) | 0.41 ( 24.13 ) | 0.41 ( 0.3 ) | -1.29 ( -1.83 ) |
| Atrioventricular block | 25 | 3.39 ( 2.29 - 5.02 ) | 3.39 ( 41.81 ) | 3.37 ( 2.43 ) | 1.75 ( 1.19 ) |
| Palpitations | 24 | 0.2 ( 0.13 - 0.3 ) | 0.2 ( 77.38 ) | 0.2 ( 0.14 ) | -2.33 ( -2.9 ) |
| Troponin i increased | 22 | 13.92 ( 9.12 - 21.24 ) | 13.91 ( 257.67 ) | 13.62 ( 9.56 ) | 3.77 ( 3.16 ) |
| Ventricular tachycardia | 22 | 1.51 ( 1 - 2.3 ) | 1.51 ( 3.83 ) | 1.51 ( 1.07 ) | 0.6 ( -0.01 ) |
| Angina pectoris | 20 | 0.78 ( 0.5 - 1.21 ) | 0.78 ( 1.25 ) | 0.78 ( 0.54 ) | -0.36 ( -0.99 ) |
| Stress cardiomyopathy | 20 | 2.99 ( 1.93 - 4.65 ) | 2.99 ( 26.43 ) | 2.98 ( 2.07 ) | 1.58 ( 0.95 ) |
| Supraventricular tachycardia | 19 | 2.23 ( 1.42 - 3.5 ) | 2.23 ( 12.87 ) | 2.23 ( 1.53 ) | 1.16 ( 0.51 ) |
| Bradycardia | 19 | 0.35 ( 0.22 - 0.55 ) | 0.35 ( 22.96 ) | 0.35 ( 0.24 ) | -1.51 ( -2.16 ) |
| Atrial flutter | 17 | 2.18 ( 1.35 - 3.5 ) | 2.18 ( 10.77 ) | 2.17 ( 1.46 ) | 1.12 ( 0.44 ) |
| Cardiac failure acute | 17 | 2.28 ( 1.42 - 3.67 ) | 2.28 ( 12.17 ) | 2.27 ( 1.53 ) | 1.19 ( 0.5 ) |
| Cardiac dysfunction | 16 | 3.22 ( 1.97 - 5.27 ) | 3.22 ( 24.37 ) | 3.21 ( 2.13 ) | 1.68 ( 0.98 ) |
| Autoimmune myocarditis | 15 | 51.16 ( 30.19 - 86.69 ) | 51.14 ( 678.93 ) | 47.16 ( 30.33 ) | 5.56 ( 4.81 ) |
| Electrocardiogram qt prolonged | 15 | 0.38 ( 0.23 - 0.62 ) | 0.38 ( 15.57 ) | 0.38 ( 0.25 ) | -1.41 ( -2.13 ) |
| Cardiotoxicity | 14 | 1.34 ( 0.79 - 2.26 ) | 1.34 ( 1.2 ) | 1.34 ( 0.86 ) | 0.42 ( -0.32 ) |
| Coronary artery disease | 12 | 0.6 ( 0.34 - 1.06 ) | 0.6 ( 3.11 ) | 0.6 ( 0.38 ) | -0.73 ( -1.53 ) |
| Ejection fraction decreased | 12 | 0.72 ( 0.41 - 1.27 ) | 0.72 ( 1.31 ) | 0.72 ( 0.45 ) | -0.47 ( -1.28 ) |
| Ventricular fibrillation | 11 | 1.18 ( 0.65 - 2.14 ) | 1.18 ( 0.31 ) | 1.18 ( 0.72 ) | 0.24 ( -0.59 ) |
| Troponin t increased | 10 | 7.96 ( 4.27 - 14.86 ) | 7.96 ( 60.08 ) | 7.87 ( 4.67 ) | 2.98 ( 2.1 ) |
| Cardiovascular disorder | 10 | 0.69 ( 0.37 - 1.28 ) | 0.69 ( 1.43 ) | 0.69 ( 0.41 ) | -0.54 ( -1.41 ) |
| Acute coronary syndrome | 9 | 1.17 ( 0.61 - 2.26 ) | 1.17 ( 0.23 ) | 1.17 ( 0.68 ) | 0.23 ( -0.68 ) |
| Bundle branch block right | 8 | 2.25 ( 1.12 - 4.5 ) | 2.25 ( 5.52 ) | 2.24 ( 1.25 ) | 1.17 ( 0.2 ) |
| Coronary artery stenosis | 8 | 2.35 ( 1.17 - 4.71 ) | 2.35 ( 6.18 ) | 2.34 ( 1.31 ) | 1.23 ( 0.27 ) |
| Sinus bradycardia | 7 | 0.77 ( 0.37 - 1.62 ) | 0.77 ( 0.47 ) | 0.77 ( 0.41 ) | -0.37 ( -1.4 ) |
| Endocarditis | 7 | 1.33 ( 0.63 - 2.79 ) | 1.33 ( 0.56 ) | 1.33 ( 0.71 ) | 0.41 ( -0.61 ) |
| Myocardial necrosis marker increased | 7 | 3.3 ( 1.57 - 6.94 ) | 3.3 ( 11.17 ) | 3.29 ( 1.77 ) | 1.72 ( 0.69 ) |
| Ventricular arrhythmia | 7 | 1.81 ( 0.86 - 3.8 ) | 1.81 ( 2.52 ) | 1.8 ( 0.97 ) | 0.85 ( -0.17 ) |
| Ventricular extrasystoles | 7 | 0.83 ( 0.4 - 1.75 ) | 0.83 ( 0.23 ) | 0.83 ( 0.45 ) | -0.26 ( -1.28 ) |
| Left ventricular dysfunction | 6 | 0.88 ( 0.4 - 1.97 ) | 0.88 ( 0.09 ) | 0.88 ( 0.45 ) | -0.18 ( -1.27 ) |
| Atrioventricular block second degree | 5 | 1.86 ( 0.77 - 4.47 ) | 1.86 ( 1.97 ) | 1.85 ( 0.89 ) | 0.89 ( -0.29 ) |
| Ischaemic cardiomyopathy | 5 | 2.58 ( 1.07 - 6.2 ) | 2.57 ( 4.8 ) | 2.57 ( 1.23 ) | 1.36 ( 0.18 ) |
| Myocardial ischaemia | 5 | 0.61 ( 0.25 - 1.47 ) | 0.61 ( 1.25 ) | 0.61 ( 0.29 ) | -0.71 ( -1.89 ) |
| Cardiomegaly | 5 | 0.55 ( 0.23 - 1.31 ) | 0.55 ( 1.88 ) | 0.55 ( 0.26 ) | -0.87 ( -2.05 ) |
| Diastolic dysfunction | 5 | 1.79 ( 0.74 - 4.3 ) | 1.79 ( 1.74 ) | 1.79 ( 0.86 ) | 0.84 ( -0.34 ) |
| Sinus node dysfunction | 5 | 1.92 ( 0.8 - 4.61 ) | 1.92 ( 2.19 ) | 1.91 ( 0.92 ) | 0.94 ( -0.24 ) |
| Bundle branch block left | 4 | 1.11 ( 0.42 - 2.97 ) | 1.11 ( 0.05 ) | 1.11 ( 0.49 ) | 0.15 ( -1.14 ) |
| Coronary artery occlusion | 4 | 0.39 ( 0.15 - 1.04 ) | 0.39 ( 3.85 ) | 0.39 ( 0.17 ) | -1.36 ( -2.65 ) |
| Left ventricular failure | 4 | 1.19 ( 0.45 - 3.17 ) | 1.19 ( 0.12 ) | 1.19 ( 0.52 ) | 0.25 ( -1.04 ) |
| Pleuropericarditis | 4 | 10.84 ( 4.03 - 29.13 ) | 10.84 ( 35.07 ) | 10.66 ( 4.66 ) | 3.41 ( 2.11 ) |
| Carditis | 4 | 9.53 ( 3.55 - 25.6 ) | 9.53 ( 30.06 ) | 9.4 ( 4.11 ) | 3.23 ( 1.93 ) |
| Ventricular dysfunction | 4 | 2.63 ( 0.99 - 7.03 ) | 2.63 ( 4.03 ) | 2.62 ( 1.15 ) | 1.39 ( 0.1 ) |
| Immune-mediated pericarditis | 4 | 76.56 ( 27.02 - 216.88 ) | 76.55 ( 264.17 ) | 67.92 ( 28.42 ) | 6.09 ( 4.71 ) |
| Mitral valve incompetence | 4 | 0.48 ( 0.18 - 1.28 ) | 0.48 ( 2.27 ) | 0.48 ( 0.21 ) | -1.06 ( -2.35 ) |
| Angina unstable | 3 | 0.62 ( 0.2 - 1.93 ) | 0.62 ( 0.69 ) | 0.62 ( 0.24 ) | -0.69 ( -2.13 ) |
| Electrocardiogram qrs complex prolonged | 3 | 0.57 ( 0.18 - 1.77 ) | 0.57 ( 0.97 ) | 0.57 ( 0.22 ) | -0.81 ( -2.25 ) |
| Autoimmune pericarditis | 3 | 177.99 ( 48.98 - 646.76 ) | 177.98 ( 406.13 ) | 137.14 ( 46.59 ) | 7.1 ( 5.46 ) |
| Pulseless electrical activity | 3 | 0.63 ( 0.2 - 1.95 ) | 0.63 ( 0.65 ) | 0.63 ( 0.24 ) | -0.67 ( -2.11 ) |
| Cardiac failure chronic | 3 | 0.56 ( 0.18 - 1.73 ) | 0.56 ( 1.04 ) | 0.56 ( 0.22 ) | -0.84 ( -2.28 ) |
| Torsade de pointes | 3 | 0.42 ( 0.13 - 1.29 ) | 0.42 ( 2.44 ) | 0.42 ( 0.16 ) | -1.26 ( -2.7 ) |
| Atrial thrombosis | 3 | 1.29 ( 0.42 - 4.02 ) | 1.29 ( 0.2 ) | 1.29 ( 0.5 ) | 0.37 ( -1.07 ) |
| Arteriosclerosis coronary artery | 3 | 0.58 ( 0.19 - 1.81 ) | 0.58 ( 0.89 ) | 0.58 ( 0.23 ) | -0.78 ( -2.22 ) |
| Aortic valve incompetence | 3 | 0.98 ( 0.32 - 3.05 ) | 0.98 ( 0 ) | 0.98 ( 0.38 ) | -0.03 ( -1.47 ) |
| Myopericarditis | 3 | 4.93 ( 1.58 - 15.36 ) | 4.93 ( 9.32 ) | 4.9 ( 1.89 ) | 2.29 ( 0.84 ) |
| Electrocardiogram st segment elevation | 3 | 1.11 ( 0.36 - 3.43 ) | 1.11 ( 0.03 ) | 1.11 ( 0.43 ) | 0.14 ( -1.3 ) |
| Conduction disorder | 2 | 1.39 ( 0.35 - 5.56 ) | 1.39 ( 0.22 ) | 1.39 ( 0.43 ) | 0.47 ( -1.2 ) |
| Prinzmetal angina | 2 | 1.95 ( 0.49 - 7.82 ) | 1.95 ( 0.92 ) | 1.95 ( 0.61 ) | 0.96 ( -0.71 ) |
| Pericardial effusion malignant | 2 | 11.87 ( 2.93 - 48.11 ) | 11.87 ( 19.51 ) | 11.65 ( 3.61 ) | 3.54 ( 1.85 ) |
| Cardiac flutter | 2 | 0.3 ( 0.07 - 1.19 ) | 0.3 ( 3.32 ) | 0.3 ( 0.09 ) | -1.75 ( -3.42 ) |
| Pericardial disease | 2 | 7.02 ( 1.74 - 28.31 ) | 7.02 ( 10.21 ) | 6.95 ( 2.16 ) | 2.8 ( 1.12 ) |
| Trifascicular block | 2 | 15.82 ( 3.88 - 64.43 ) | 15.82 ( 27.05 ) | 15.44 ( 4.77 ) | 3.95 ( 2.25 ) |
| Cardiopulmonary failure | 2 | 0.6 ( 0.15 - 2.41 ) | 0.6 ( 0.52 ) | 0.6 ( 0.19 ) | -0.73 ( -2.4 ) |
| Cardiovascular insufficiency | 2 | 1.22 ( 0.31 - 4.89 ) | 1.22 ( 0.08 ) | 1.22 ( 0.38 ) | 0.29 ( -1.38 ) |
| Electrocardiogram change | 2 | 3.61 ( 0.9 - 14.48 ) | 3.61 ( 3.74 ) | 3.59 ( 1.12 ) | 1.84 ( 0.17 ) |
| Pericarditis constrictive | 2 | 4.82 ( 1.2 - 19.4 ) | 4.82 ( 6.01 ) | 4.79 ( 1.5 ) | 2.26 ( 0.58 ) |
| Atrial tachycardia | 2 | 0.94 ( 0.24 - 3.78 ) | 0.94 ( 0.01 ) | 0.94 ( 0.3 ) | -0.08 ( -1.75 ) |
| Cardiac valve disease | 2 | 0.38 ( 0.1 - 1.53 ) | 0.38 ( 1.99 ) | 0.38 ( 0.12 ) | -1.38 ( -3.05 ) |
| Pericarditis malignant | 2 | 14.13 ( 3.48 - 57.42 ) | 14.13 ( 23.83 ) | 13.82 ( 4.27 ) | 3.79 ( 2.09 ) |
| Cardiac hypertrophy | 2 | 2.12 ( 0.53 - 8.49 ) | 2.12 ( 1.18 ) | 2.11 ( 0.66 ) | 1.08 ( -0.59 ) |
| Cardiac sarcoidosis | 2 | 8.36 ( 2.07 - 33.74 ) | 8.36 ( 12.77 ) | 8.25 ( 2.57 ) | 3.05 ( 1.36 ) |
| Electrocardiogram st segment depression | 2 | 1.19 ( 0.3 - 4.76 ) | 1.19 ( 0.06 ) | 1.19 ( 0.37 ) | 0.25 ( -1.42 ) |
| Ventricular hypokinesia | 2 | 0.68 ( 0.17 - 2.73 ) | 0.68 ( 0.3 ) | 0.68 ( 0.21 ) | -0.55 ( -2.22 ) |
| Left ventricular hypertrophy | 2 | 0.55 ( 0.14 - 2.2 ) | 0.55 ( 0.74 ) | 0.55 ( 0.17 ) | -0.86 ( -2.53 ) |
| Electrocardiogram abnormal | 1 | 0.14 ( 0.02 - 0.98 ) | 0.14 ( 5.37 ) | 0.14 ( 0.03 ) | -2.85 ( -4.89 ) |
| Coronary artery insufficiency | 1 | 4.53 ( 0.63 - 32.39 ) | 4.53 ( 2.73 ) | 4.5 ( 0.87 ) | 2.17 ( 0.12 ) |
| Cardiac asthma | 1 | 7.7 ( 1.07 - 55.4 ) | 7.7 ( 5.76 ) | 7.62 ( 1.46 ) | 2.93 ( 0.86 ) |
| Atrial enlargement | 1 | 2.58 ( 0.36 - 18.39 ) | 2.58 ( 0.96 ) | 2.57 ( 0.5 ) | 1.36 ( -0.69 ) |
| Supraventricular extrasystoles | 1 | 0.38 ( 0.05 - 2.68 ) | 0.38 ( 1.03 ) | 0.38 ( 0.07 ) | -1.4 ( -3.45 ) |
| Extrasystoles | 1 | 0.16 ( 0.02 - 1.13 ) | 0.16 ( 4.47 ) | 0.16 ( 0.03 ) | -2.65 ( -4.7 ) |
| Myocardial fibrosis | 1 | 1.21 ( 0.17 - 8.58 ) | 1.21 ( 0.04 ) | 1.21 ( 0.23 ) | 0.27 ( -1.77 ) |
| Acute left ventricular failure | 1 | 1.23 ( 0.17 - 8.72 ) | 1.23 ( 0.04 ) | 1.23 ( 0.24 ) | 0.29 ( -1.75 ) |
| Pericardial haemorrhage | 1 | 0.42 ( 0.06 - 2.96 ) | 0.42 ( 0.82 ) | 0.42 ( 0.08 ) | -1.26 ( -3.3 ) |
| Mitral valve disease | 1 | 0.68 ( 0.1 - 4.85 ) | 0.68 ( 0.15 ) | 0.68 ( 0.13 ) | -0.55 ( -2.59 ) |
| Tricuspid valve disease | 1 | 2.75 ( 0.39 - 19.59 ) | 2.75 ( 1.11 ) | 2.74 ( 0.53 ) | 1.45 ( -0.6 ) |
| Heart alternation | 1 | 10.79 ( 1.49 - 77.95 ) | 10.79 ( 8.72 ) | 10.61 ( 2.03 ) | 3.41 ( 1.33 ) |
| Cardiovascular deconditioning | 1 | 31.23 ( 4.18 - 233.26 ) | 31.23 ( 27.79 ) | 29.71 ( 5.52 ) | 4.89 ( 2.76 ) |
| Nodal rhythm | 1 | 0.96 ( 0.13 - 6.79 ) | 0.96 ( 0 ) | 0.96 ( 0.19 ) | -0.07 ( -2.11 ) |
| Aortic valve stenosis | 1 | 0.57 ( 0.08 - 4.07 ) | 0.57 ( 0.32 ) | 0.57 ( 0.11 ) | -0.8 ( -2.85 ) |
| Cardio-respiratory distress | 1 | 4.06 ( 0.57 - 29.04 ) | 4.06 ( 2.29 ) | 4.04 ( 0.78 ) | 2.02 ( -0.04 ) |
| Nodal arrhythmia | 1 | 1.7 ( 0.24 - 12.1 ) | 1.7 ( 0.29 ) | 1.7 ( 0.33 ) | 0.76 ( -1.28 ) |
| Wellens' syndrome | 1 | 21.97 ( 2.99 - 161.71 ) | 21.97 ( 19.3 ) | 21.22 ( 3.99 ) | 4.41 ( 2.3 ) |
| Bundle branch block | 1 | 1.35 ( 0.19 - 9.59 ) | 1.35 ( 0.09 ) | 1.35 ( 0.26 ) | 0.43 ( -1.61 ) |
| Electrocardiogram q wave abnormal | 1 | 5.2 ( 0.73 - 37.27 ) | 5.2 ( 3.37 ) | 5.17 ( 1 ) | 2.37 ( 0.31 ) |
| Tachycardia induced cardiomyopathy | 1 | 5.49 ( 0.77 - 39.35 ) | 5.49 ( 3.64 ) | 5.45 ( 1.05 ) | 2.45 ( 0.39 ) |
| Subendocardial ischaemia | 1 | 12.62 ( 1.74 - 91.49 ) | 12.62 ( 10.48 ) | 12.38 ( 2.36 ) | 3.63 ( 1.55 ) |
| Kounis syndrome | 1 | 0.36 ( 0.05 - 2.58 ) | 0.36 ( 1.11 ) | 0.36 ( 0.07 ) | -1.46 ( -3.5 ) |
| Aortic valve disease | 1 | 0.66 ( 0.09 - 4.72 ) | 0.66 ( 0.17 ) | 0.66 ( 0.13 ) | -0.59 ( -2.63 ) |
| Electrocardiogram t wave abnormal | 1 | 0.89 ( 0.12 - 6.3 ) | 0.89 ( 0.01 ) | 0.89 ( 0.17 ) | -0.18 ( -2.22 ) |
| Myocardial necrosis | 1 | 3.04 ( 0.43 - 21.71 ) | 3.04 ( 1.36 ) | 3.03 ( 0.59 ) | 1.6 ( -0.45 ) |
| Ejection fraction abnormal | 1 | 0.62 ( 0.09 - 4.38 ) | 0.62 ( 0.24 ) | 0.62 ( 0.12 ) | -0.7 ( -2.74 ) |
| Pulmonary valve incompetence | 1 | 0.99 ( 0.14 - 7.03 ) | 0.99 ( 0 ) | 0.99 ( 0.19 ) | -0.02 ( -2.06 ) |
| Ventricular tachyarrhythmia | 1 | 2.64 ( 0.37 - 18.8 ) | 2.64 ( 1.01 ) | 2.63 ( 0.51 ) | 1.39 ( -0.65 ) |
| Acute right ventricular failure | 1 | 4.86 ( 0.68 - 34.8 ) | 4.86 ( 3.04 ) | 4.83 ( 0.93 ) | 2.27 ( 0.22 ) |
| Right ventricular failure | 1 | 0.13 ( 0.02 - 0.94 ) | 0.13 ( 5.67 ) | 0.13 ( 0.03 ) | -2.91 ( -4.95 ) |
| Arteriospasm coronary | 1 | 0.27 ( 0.04 - 1.89 ) | 0.27 ( 2.03 ) | 0.27 ( 0.05 ) | -1.91 ( -3.95 ) |
| Cardiorenal syndrome | 1 | 1.29 ( 0.18 - 9.2 ) | 1.29 ( 0.07 ) | 1.29 ( 0.25 ) | 0.37 ( -1.68 ) |
| Cardiac ventricular thrombosis | 1 | 0.7 ( 0.1 - 4.96 ) | 0.7 ( 0.13 ) | 0.7 ( 0.14 ) | -0.52 ( -2.56 ) |
| Heart valve incompetence | 1 | 0.33 ( 0.05 - 2.36 ) | 0.33 ( 1.35 ) | 0.33 ( 0.06 ) | -1.59 ( -3.63 ) |
| Tachyarrhythmia | 1 | 0.44 ( 0.06 - 3.1 ) | 0.44 ( 0.73 ) | 0.44 ( 0.08 ) | -1.19 ( -3.24 ) |
| Silent myocardial infarction | 1 | 2.58 ( 0.36 - 18.39 ) | 2.58 ( 0.96 ) | 2.57 ( 0.5 ) | 1.36 ( -0.69 ) |
| Myocardial injury | 1 | 1.01 ( 0.14 - 7.21 ) | 1.01 ( 0 ) | 1.01 ( 0.2 ) | 0.02 ( -2.02 ) |
| Arrhythmia supraventricular | 1 | 1.16 ( 0.16 - 8.26 ) | 1.16 ( 0.02 ) | 1.16 ( 0.22 ) | 0.21 ( -1.83 ) |
| Dilated cardiomyopathy | 1 | 1.12 ( 0.16 - 7.93 ) | 1.12 ( 0.01 ) | 1.11 ( 0.22 ) | 0.16 ( -1.89 ) |
| Troponin abnormal | 1 | 6.67 ( 0.93 - 47.85 ) | 6.67 ( 4.76 ) | 6.6 ( 1.27 ) | 2.72 ( 0.66 ) |
| Toxic cardiomyopathy | 1 | 3.68 ( 0.52 - 26.32 ) | 3.68 ( 1.94 ) | 3.67 ( 0.71 ) | 1.88 ( -0.18 ) |

Abbreviation: ROR, reporting odds ratio; PRR, proportional reporting ratio; EBGM, empirical Bayesian geometric mean; EBGM05, the lower limit of the 95% CI of EBGM; IC, information component; IC025, the lower limit of the 95% CI of the IC; CI, confidence interval; PT, preferred term.

**Supplementary Table S9:**

**Signal strength of cardiac adverse events associated with dual ICIs in melanoma patients in the FAERS database**

| PT | Numbers | ROR(95%Cl) | PRR(X²) | EBGM(EBGM05) | IC(IC025) |
| --- | --- | --- | --- | --- | --- |
| Myocarditis | 133 | 26.67 ( 22.46 - 31.67 ) | 26.54 ( 3212.14 ) | 26.09 ( 22.6 ) | 4.71 ( 4.45 ) |
| Atrial fibrillation | 69 | 1.68 ( 1.33 - 2.13 ) | 1.68 ( 18.98 ) | 1.68 ( 1.38 ) | 0.75 ( 0.4 ) |
| Immune-mediated myocarditis | 64 | 103.44 ( 80.26 - 133.31 ) | 103.2 ( 6054.76 ) | 96.53 ( 78.07 ) | 6.59 ( 6.22 ) |
| Cardiac failure | 33 | 0.98 ( 0.69 - 1.37 ) | 0.98 ( 0.02 ) | 0.98 ( 0.73 ) | -0.03 ( -0.53 ) |
| Pericardial effusion | 32 | 3.44 ( 2.43 - 4.87 ) | 3.44 ( 55.16 ) | 3.43 ( 2.57 ) | 1.78 ( 1.28 ) |
| Myocardial infarction | 30 | 0.56 ( 0.39 - 0.8 ) | 0.56 ( 10.37 ) | 0.56 ( 0.42 ) | -0.84 ( -1.35 ) |
| Troponin increased | 23 | 7.75 ( 5.14 - 11.68 ) | 7.74 ( 134.41 ) | 7.71 ( 5.47 ) | 2.95 ( 2.36 ) |
| Tachycardia | 22 | 0.62 ( 0.41 - 0.94 ) | 0.62 ( 5.23 ) | 0.62 ( 0.44 ) | -0.7 ( -1.3 ) |
| Cardiac arrest | 19 | 0.65 ( 0.41 - 1.01 ) | 0.65 ( 3.7 ) | 0.65 ( 0.44 ) | -0.63 ( -1.28 ) |
| Cardiac disorder | 18 | 0.49 ( 0.31 - 0.78 ) | 0.49 ( 9.37 ) | 0.49 ( 0.34 ) | -1.02 ( -1.68 ) |
| Arrhythmia | 15 | 0.83 ( 0.5 - 1.37 ) | 0.83 ( 0.55 ) | 0.83 ( 0.54 ) | -0.28 ( -1 ) |
| Cardiomyopathy | 14 | 2.66 ( 1.57 - 4.49 ) | 2.66 ( 14.47 ) | 2.66 ( 1.71 ) | 1.41 ( 0.66 ) |
| Pericarditis | 14 | 1.86 ( 1.1 - 3.14 ) | 1.86 ( 5.52 ) | 1.85 ( 1.2 ) | 0.89 ( 0.15 ) |
| Acute myocardial infarction | 14 | 1.28 ( 0.76 - 2.16 ) | 1.28 ( 0.85 ) | 1.28 ( 0.82 ) | 0.35 ( -0.39 ) |
| Sinus tachycardia | 13 | 2.59 ( 1.51 - 4.47 ) | 2.59 ( 12.71 ) | 2.59 ( 1.64 ) | 1.37 ( 0.6 ) |
| Cardiogenic shock | 12 | 2.04 ( 1.16 - 3.59 ) | 2.04 ( 6.35 ) | 2.04 ( 1.27 ) | 1.03 ( 0.23 ) |
| Ventricular tachycardia | 11 | 1.88 ( 1.04 - 3.4 ) | 1.88 ( 4.56 ) | 1.88 ( 1.15 ) | 0.91 ( 0.08 ) |
| Atrioventricular block complete | 10 | 4.08 ( 2.19 - 7.59 ) | 4.08 ( 23.19 ) | 4.07 ( 2.42 ) | 2.03 ( 1.15 ) |
| Palpitations | 9 | 0.19 ( 0.1 - 0.36 ) | 0.19 ( 32.08 ) | 0.19 ( 0.11 ) | -2.42 ( -3.34 ) |
| Troponin i increased | 9 | 14 ( 7.26 - 26.99 ) | 13.99 ( 107.57 ) | 13.87 ( 8.01 ) | 3.79 ( 2.88 ) |
| Coronary artery disease | 8 | 1 ( 0.5 - 2.01 ) | 1 ( 0 ) | 1 ( 0.56 ) | 0.01 ( -0.96 ) |
| Supraventricular tachycardia | 8 | 2.34 ( 1.17 - 4.68 ) | 2.34 ( 6.11 ) | 2.33 ( 1.31 ) | 1.22 ( 0.26 ) |
| Cardiac tamponade | 8 | 4.04 ( 2.02 - 8.09 ) | 4.04 ( 18.25 ) | 4.03 ( 2.26 ) | 2.01 ( 1.05 ) |
| Cardio-respiratory arrest | 7 | 0.47 ( 0.22 - 0.99 ) | 0.47 ( 4.19 ) | 0.47 ( 0.25 ) | -1.09 ( -2.11 ) |
| Atrial flutter | 6 | 1.91 ( 0.86 - 4.25 ) | 1.91 ( 2.6 ) | 1.91 ( 0.98 ) | 0.93 ( -0.16 ) |
| Angina pectoris | 6 | 0.58 ( 0.26 - 1.3 ) | 0.58 ( 1.8 ) | 0.58 ( 0.3 ) | -0.78 ( -1.87 ) |
| Atrioventricular block | 6 | 2.02 ( 0.91 - 4.49 ) | 2.02 ( 3.07 ) | 2.02 ( 1.03 ) | 1.01 ( -0.08 ) |
| Bradycardia | 6 | 0.28 ( 0.12 - 0.61 ) | 0.28 ( 11.46 ) | 0.28 ( 0.14 ) | -1.86 ( -2.95 ) |
| Autoimmune myocarditis | 6 | 48.49 ( 21.5 - 109.36 ) | 48.48 ( 270.14 ) | 46.97 ( 23.79 ) | 5.55 ( 4.44 ) |
| Cardiotoxicity | 6 | 1.43 ( 0.64 - 3.18 ) | 1.43 ( 0.77 ) | 1.43 ( 0.73 ) | 0.51 ( -0.58 ) |
| Sinus bradycardia | 5 | 1.37 ( 0.57 - 3.3 ) | 1.37 ( 0.5 ) | 1.37 ( 0.66 ) | 0.46 ( -0.72 ) |
| Cardiac failure congestive | 5 | 0.18 ( 0.08 - 0.44 ) | 0.18 ( 18.51 ) | 0.18 ( 0.09 ) | -2.46 ( -3.64 ) |
| Stress cardiomyopathy | 5 | 1.86 ( 0.77 - 4.47 ) | 1.86 ( 1.98 ) | 1.86 ( 0.89 ) | 0.89 ( -0.29 ) |
| Ejection fraction decreased | 5 | 0.75 ( 0.31 - 1.79 ) | 0.75 ( 0.43 ) | 0.75 ( 0.36 ) | -0.42 ( -1.6 ) |
| Endocarditis | 4 | 1.89 ( 0.71 - 5.04 ) | 1.89 ( 1.67 ) | 1.89 ( 0.83 ) | 0.92 ( -0.38 ) |
| Myocardial necrosis marker increased | 4 | 4.69 ( 1.76 - 12.52 ) | 4.69 ( 11.58 ) | 4.68 ( 2.06 ) | 2.23 ( 0.93 ) |
| Ventricular arrhythmia | 4 | 2.57 ( 0.96 - 6.86 ) | 2.57 ( 3.83 ) | 2.57 ( 1.13 ) | 1.36 ( 0.07 ) |
| Atrioventricular block second degree | 4 | 3.7 ( 1.39 - 9.87 ) | 3.7 ( 7.86 ) | 3.69 ( 1.62 ) | 1.88 ( 0.59 ) |
| Troponin t increased | 4 | 7.88 ( 2.95 - 21.04 ) | 7.88 ( 23.88 ) | 7.84 ( 3.44 ) | 2.97 ( 1.68 ) |
| Myocardial ischaemia | 3 | 0.91 ( 0.29 - 2.83 ) | 0.91 ( 0.03 ) | 0.91 ( 0.35 ) | -0.13 ( -1.58 ) |
| Cardiomegaly | 3 | 0.82 ( 0.26 - 2.53 ) | 0.82 ( 0.12 ) | 0.82 ( 0.32 ) | -0.29 ( -1.73 ) |
| Left ventricular failure | 3 | 2.22 ( 0.72 - 6.89 ) | 2.22 ( 2.01 ) | 2.22 ( 0.86 ) | 1.15 ( -0.3 ) |
| Bundle branch block right | 3 | 2.1 ( 0.68 - 6.5 ) | 2.1 ( 1.72 ) | 2.09 ( 0.81 ) | 1.07 ( -0.38 ) |
| Electrocardiogram qt prolonged | 3 | 0.19 ( 0.06 - 0.58 ) | 0.19 ( 10.6 ) | 0.19 ( 0.07 ) | -2.42 ( -3.86 ) |
| Left ventricular dysfunction | 3 | 1.1 ( 0.35 - 3.41 ) | 1.1 ( 0.03 ) | 1.1 ( 0.43 ) | 0.14 ( -1.31 ) |
| Acute coronary syndrome | 3 | 0.97 ( 0.31 - 3.02 ) | 0.97 ( 0 ) | 0.97 ( 0.38 ) | -0.04 ( -1.48 ) |
| Cardiac sarcoidosis | 3 | 31.46 ( 10.03 - 98.74 ) | 31.46 ( 86.63 ) | 30.83 ( 11.84 ) | 4.95 ( 3.48 ) |
| Ventricular extrasystoles | 3 | 0.89 ( 0.29 - 2.76 ) | 0.89 ( 0.04 ) | 0.89 ( 0.34 ) | -0.17 ( -1.61 ) |
| Arteriosclerosis coronary artery | 3 | 1.45 ( 0.47 - 4.51 ) | 1.45 ( 0.42 ) | 1.45 ( 0.56 ) | 0.54 ( -0.91 ) |
| Coronary artery occlusion | 2 | 0.48 ( 0.12 - 1.94 ) | 0.48 ( 1.1 ) | 0.48 ( 0.15 ) | -1.05 ( -2.71 ) |
| Angina unstable | 2 | 1.03 ( 0.26 - 4.13 ) | 1.03 ( 0 ) | 1.03 ( 0.32 ) | 0.05 ( -1.62 ) |
| Pleuropericarditis | 2 | 13.38 ( 3.33 - 53.85 ) | 13.38 ( 22.71 ) | 13.27 ( 4.14 ) | 3.73 ( 2.05 ) |
| Electrocardiogram change | 2 | 8.99 ( 2.24 - 36.1 ) | 8.99 ( 14.11 ) | 8.94 ( 2.79 ) | 3.16 ( 1.49 ) |
| Ventricular fibrillation | 2 | 0.54 ( 0.13 - 2.14 ) | 0.54 ( 0.81 ) | 0.54 ( 0.17 ) | -0.9 ( -2.57 ) |
| Electrocardiogram st segment depression | 2 | 2.96 ( 0.74 - 11.85 ) | 2.96 ( 2.59 ) | 2.96 ( 0.93 ) | 1.56 ( -0.1 ) |
| Cardiac failure acute | 2 | 0.67 ( 0.17 - 2.66 ) | 0.67 ( 0.33 ) | 0.67 ( 0.21 ) | -0.59 ( -2.25 ) |
| Immune-mediated pericarditis | 2 | 89.62 ( 21.5 - 373.5 ) | 89.61 ( 165.23 ) | 84.55 ( 25.61 ) | 6.4 ( 4.67 ) |
| Electrocardiogram abnormal | 1 | 0.34 ( 0.05 - 2.45 ) | 0.34 ( 1.25 ) | 0.34 ( 0.07 ) | -1.54 ( -3.58 ) |
| Conduction disorder | 1 | 1.73 ( 0.24 - 12.29 ) | 1.73 ( 0.31 ) | 1.73 ( 0.33 ) | 0.79 ( -1.25 ) |
| Ischaemic cardiomyopathy | 1 | 1.28 ( 0.18 - 9.09 ) | 1.28 ( 0.06 ) | 1.28 ( 0.25 ) | 0.35 ( -1.69 ) |
| Cardiac flutter | 1 | 0.37 ( 0.05 - 2.63 ) | 0.37 ( 1.07 ) | 0.37 ( 0.07 ) | -1.43 ( -3.47 ) |
| Bundle branch block left | 1 | 0.69 ( 0.1 - 4.91 ) | 0.69 ( 0.14 ) | 0.69 ( 0.13 ) | -0.53 ( -2.57 ) |
| Extrasystoles | 1 | 0.4 ( 0.06 - 2.81 ) | 0.4 ( 0.93 ) | 0.4 ( 0.08 ) | -1.34 ( -3.38 ) |
| Pericardial disease | 1 | 8.7 ( 1.22 - 62.11 ) | 8.7 ( 6.77 ) | 8.65 ( 1.67 ) | 3.11 ( 1.06 ) |
| Myocardial fibrosis | 1 | 3.01 ( 0.42 - 21.38 ) | 3.01 ( 1.34 ) | 3 ( 0.58 ) | 1.59 ( -0.46 ) |
| Trifascicular block | 1 | 19.46 ( 2.71 - 139.92 ) | 19.46 ( 17.28 ) | 19.22 ( 3.69 ) | 4.26 ( 2.2 ) |
| Acute left ventricular failure | 1 | 3.06 ( 0.43 - 21.73 ) | 3.05 ( 1.38 ) | 3.05 ( 0.59 ) | 1.61 ( -0.44 ) |
| Mitral valve disease | 1 | 1.7 ( 0.24 - 12.09 ) | 1.7 ( 0.29 ) | 1.7 ( 0.33 ) | 0.77 ( -1.28 ) |
| Tricuspid valve disease | 1 | 6.85 ( 0.96 - 48.82 ) | 6.85 ( 4.97 ) | 6.82 ( 1.32 ) | 2.77 ( 0.72 ) |
| Electrocardiogram qrs complex prolonged | 1 | 0.47 ( 0.07 - 3.36 ) | 0.47 ( 0.59 ) | 0.47 ( 0.09 ) | -1.08 ( -3.12 ) |
| Heart alternation | 1 | 26.88 ( 3.72 - 194.29 ) | 26.88 ( 24.48 ) | 26.42 ( 5.05 ) | 4.72 ( 2.65 ) |
| Cardiovascular disorder | 1 | 0.17 ( 0.02 - 1.21 ) | 0.17 ( 4.02 ) | 0.17 ( 0.03 ) | -2.55 ( -4.59 ) |
| Cardiovascular deconditioning | 1 | 77.82 ( 10.42 - 581.38 ) | 77.82 ( 72.04 ) | 73.98 ( 13.75 ) | 6.21 ( 4.07 ) |
| Atrial tachycardia | 1 | 1.18 ( 0.17 - 8.36 ) | 1.18 ( 0.03 ) | 1.18 ( 0.23 ) | 0.23 ( -1.81 ) |
| Cardiac valve disease | 1 | 0.48 ( 0.07 - 3.39 ) | 0.48 ( 0.57 ) | 0.48 ( 0.09 ) | -1.07 ( -3.11 ) |
| Torsade de pointes | 1 | 0.35 ( 0.05 - 2.46 ) | 0.35 ( 1.23 ) | 0.35 ( 0.07 ) | -1.53 ( -3.57 ) |
| Cardiac dysfunction | 1 | 0.5 ( 0.07 - 3.55 ) | 0.5 ( 0.5 ) | 0.5 ( 0.1 ) | -1 ( -3.04 ) |
| Aortic valve incompetence | 1 | 0.81 ( 0.11 - 5.78 ) | 0.81 ( 0.04 ) | 0.81 ( 0.16 ) | -0.3 ( -2.34 ) |
| Sinus node dysfunction | 1 | 0.95 ( 0.13 - 6.77 ) | 0.95 ( 0 ) | 0.95 ( 0.18 ) | -0.07 ( -2.11 ) |
| Coronary artery stenosis | 1 | 0.73 ( 0.1 - 5.18 ) | 0.73 ( 0.1 ) | 0.73 ( 0.14 ) | -0.45 ( -2.5 ) |
| Cardiac failure chronic | 1 | 0.46 ( 0.07 - 3.3 ) | 0.46 ( 0.62 ) | 0.46 ( 0.09 ) | -1.11 ( -3.15 ) |
| Electrocardiogram t wave abnormal | 1 | 2.21 ( 0.31 - 15.69 ) | 2.21 ( 0.66 ) | 2.21 ( 0.43 ) | 1.14 ( -0.9 ) |
| Myopericarditis | 1 | 4.07 ( 0.57 - 29 ) | 4.07 ( 2.31 ) | 4.06 ( 0.79 ) | 2.02 ( -0.02 ) |
| Atrial thrombosis | 1 | 1.07 ( 0.15 - 7.63 ) | 1.07 ( 0.01 ) | 1.07 ( 0.21 ) | 0.1 ( -1.94 ) |
| Cardiac ventricular thrombosis | 1 | 1.74 ( 0.24 - 12.35 ) | 1.74 ( 0.31 ) | 1.74 ( 0.34 ) | 0.8 ( -1.25 ) |
| Heart valve incompetence | 1 | 0.83 ( 0.12 - 5.87 ) | 0.83 ( 0.04 ) | 0.83 ( 0.16 ) | -0.27 ( -2.32 ) |
| Dilated cardiomyopathy | 1 | 2.78 ( 0.39 - 19.77 ) | 2.78 ( 1.14 ) | 2.78 ( 0.54 ) | 1.47 ( -0.57 ) |
| Tachyarrhythmia | 1 | 1.09 ( 0.15 - 7.74 ) | 1.09 ( 0.01 ) | 1.09 ( 0.21 ) | 0.12 ( -1.92 ) |
| Electrocardiogram st segment elevation | 1 | 0.92 ( 0.13 - 6.52 ) | 0.92 ( 0.01 ) | 0.92 ( 0.18 ) | -0.12 ( -2.17 ) |
| Pericarditis constrictive | 1 | 5.99 ( 0.84 - 42.67 ) | 5.99 ( 4.14 ) | 5.97 ( 1.15 ) | 2.58 ( 0.53 ) |
| Left ventricular hypertrophy | 1 | 0.68 ( 0.1 - 4.86 ) | 0.68 ( 0.15 ) | 0.68 ( 0.13 ) | -0.55 ( -2.59 ) |
| Myocardial injury | 1 | 2.53 ( 0.36 - 17.97 ) | 2.53 ( 0.92 ) | 2.52 ( 0.49 ) | 1.34 ( -0.71 ) |
| Mitral valve incompetence | 1 | 0.3 ( 0.04 - 2.12 ) | 0.3 ( 1.65 ) | 0.3 ( 0.06 ) | -1.75 ( -3.79 ) |

Abbreviation: ROR, reporting odds ratio; PRR, proportional reporting ratio; EBGM, empirical Bayesian geometric mean; EBGM05, the lower limit of the 95% CI of EBGM; IC, information component; IC025, the lower limit of the 95% CI of the IC; CI, confidence interval; PT, preferred term.

**Supplementary Table S10:**

**Signal strength of cardiac adverse events associated with dual ICIs in lung cancer patients in the FAERS database**

| PT | a | ROR(95%Cl) | PRR(X²) | EBGM(EBGM05) | IC(IC025) |
| --- | --- | --- | --- | --- | --- |
| Myocarditis | 53 | 21.4 ( 16.33 - 28.06 ) | 21.32 ( 1019.5 ) | 21.18 ( 16.89 ) | 4.4 ( 4.01 ) |
| Cardiac failure | 43 | 2.6 ( 1.93 - 3.51 ) | 2.59 ( 42.09 ) | 2.59 ( 2.02 ) | 1.37 ( 0.94 ) |
| Pericardial effusion | 35 | 7.68 ( 5.51 - 10.7 ) | 7.66 ( 202.2 ) | 7.64 ( 5.79 ) | 2.93 ( 2.45 ) |
| Atrial fibrillation | 33 | 1.64 ( 1.16 - 2.3 ) | 1.64 ( 8.16 ) | 1.64 ( 1.23 ) | 0.71 ( 0.21 ) |
| Immune-mediated myocarditis | 24 | 75.69 ( 50.46 - 113.53 ) | 75.55 ( 1722.48 ) | 73.73 ( 52.52 ) | 6.2 ( 5.62 ) |
| Cardiac tamponade | 19 | 19.65 ( 12.51 - 30.86 ) | 19.62 ( 333.67 ) | 19.5 ( 13.37 ) | 4.29 ( 3.64 ) |
| Cardio-respiratory arrest | 16 | 2.19 ( 1.34 - 3.58 ) | 2.19 ( 10.32 ) | 2.19 ( 1.45 ) | 1.13 ( 0.43 ) |
| Cardiac arrest | 15 | 1.04 ( 0.63 - 1.72 ) | 1.04 ( 0.02 ) | 1.04 ( 0.68 ) | 0.05 ( -0.67 ) |
| Tachycardia | 13 | 0.74 ( 0.43 - 1.28 ) | 0.74 ( 1.16 ) | 0.74 ( 0.47 ) | -0.43 ( -1.2 ) |
| Arrhythmia | 12 | 1.35 ( 0.76 - 2.37 ) | 1.35 ( 1.07 ) | 1.35 ( 0.84 ) | 0.43 ( -0.37 ) |
| Acute myocardial infarction | 12 | 2.23 ( 1.27 - 3.93 ) | 2.23 ( 8.16 ) | 2.23 ( 1.39 ) | 1.16 ( 0.36 ) |
| Myocardial infarction | 11 | 0.42 ( 0.23 - 0.75 ) | 0.42 ( 8.91 ) | 0.42 ( 0.26 ) | -1.26 ( -2.09 ) |
| Atrial flutter | 10 | 6.49 ( 3.49 - 12.08 ) | 6.49 ( 46.35 ) | 6.48 ( 3.85 ) | 2.7 ( 1.82 ) |
| Cardiac failure congestive | 9 | 0.66 ( 0.35 - 1.28 ) | 0.66 ( 1.52 ) | 0.66 ( 0.38 ) | -0.59 ( -1.5 ) |
| Atrioventricular block complete | 8 | 6.65 ( 3.32 - 13.31 ) | 6.65 ( 38.3 ) | 6.63 ( 3.71 ) | 2.73 ( 1.77 ) |
| Pericarditis | 8 | 2.16 ( 1.08 - 4.32 ) | 2.16 ( 4.98 ) | 2.16 ( 1.21 ) | 1.11 ( 0.15 ) |
| Cardiac disorder | 8 | 0.45 ( 0.22 - 0.89 ) | 0.45 ( 5.49 ) | 0.45 ( 0.25 ) | -1.16 ( -2.12 ) |
| Ventricular fibrillation | 8 | 4.37 ( 2.18 - 8.74 ) | 4.37 ( 20.73 ) | 4.36 ( 2.44 ) | 2.12 ( 1.16 ) |
| Atrioventricular block | 7 | 4.8 ( 2.29 - 10.07 ) | 4.8 ( 21 ) | 4.79 ( 2.57 ) | 2.26 ( 1.24 ) |
| Sinus tachycardia | 7 | 2.84 ( 1.36 - 5.97 ) | 2.84 ( 8.36 ) | 2.84 ( 1.53 ) | 1.51 ( 0.49 ) |
| Acute coronary syndrome | 6 | 3.97 ( 1.78 - 8.85 ) | 3.97 ( 13.32 ) | 3.97 ( 2.03 ) | 1.99 ( 0.9 ) |
| Cardiogenic shock | 6 | 2.08 ( 0.93 - 4.62 ) | 2.08 ( 3.34 ) | 2.08 ( 1.06 ) | 1.05 ( -0.04 ) |
| Cardiac failure acute | 6 | 4.08 ( 1.83 - 9.08 ) | 4.08 ( 13.91 ) | 4.07 ( 2.08 ) | 2.03 ( 0.93 ) |
| Troponin increased | 5 | 3.42 ( 1.42 - 8.22 ) | 3.42 ( 8.54 ) | 3.41 ( 1.64 ) | 1.77 ( 0.59 ) |
| Stress cardiomyopathy | 5 | 3.79 ( 1.58 - 9.11 ) | 3.79 ( 10.24 ) | 3.78 ( 1.82 ) | 1.92 ( 0.74 ) |
| Cardiovascular disorder | 5 | 1.74 ( 0.73 - 4.19 ) | 1.74 ( 1.58 ) | 1.74 ( 0.84 ) | 0.8 ( -0.38 ) |
| Cardiomyopathy | 4 | 1.55 ( 0.58 - 4.12 ) | 1.55 ( 0.77 ) | 1.55 ( 0.68 ) | 0.63 ( -0.66 ) |
| Supraventricular tachycardia | 4 | 2.38 ( 0.89 - 6.34 ) | 2.38 ( 3.19 ) | 2.38 ( 1.05 ) | 1.25 ( -0.04 ) |
| Pericarditis malignant | 4 | 147.01 ( 53.88 - 401.11 ) | 146.96 ( 552.91 ) | 140.17 ( 60.52 ) | 7.13 ( 5.8 ) |
| Coronary artery stenosis | 4 | 5.96 ( 2.23 - 15.89 ) | 5.95 ( 16.46 ) | 5.94 ( 2.62 ) | 2.57 ( 1.28 ) |
| Coronary artery disease | 3 | 0.77 ( 0.25 - 2.38 ) | 0.77 ( 0.21 ) | 0.77 ( 0.3 ) | -0.38 ( -1.83 ) |
| Troponin i increased | 3 | 9.45 ( 3.04 - 29.35 ) | 9.44 ( 22.58 ) | 9.42 ( 3.65 ) | 3.24 ( 1.79 ) |
| Autoimmune myocarditis | 3 | 48.6 ( 15.53 - 152.1 ) | 48.59 ( 137.62 ) | 47.84 ( 18.42 ) | 5.58 ( 4.12 ) |
| Carditis | 3 | 36.16 ( 11.58 - 112.9 ) | 36.15 ( 101.33 ) | 35.74 ( 13.78 ) | 5.16 ( 3.71 ) |
| Sinus node dysfunction | 3 | 5.84 ( 1.88 - 18.12 ) | 5.83 ( 12 ) | 5.83 ( 2.26 ) | 2.54 ( 1.1 ) |
| Cardiac dysfunction | 3 | 3.05 ( 0.98 - 9.48 ) | 3.05 ( 4.14 ) | 3.05 ( 1.18 ) | 1.61 ( 0.17 ) |
| Ventricular tachycardia | 3 | 1.05 ( 0.34 - 3.24 ) | 1.05 ( 0.01 ) | 1.05 ( 0.41 ) | 0.06 ( -1.38 ) |
| Bundle branch block right | 3 | 4.27 ( 1.38 - 13.25 ) | 4.27 ( 7.5 ) | 4.26 ( 1.65 ) | 2.09 ( 0.65 ) |
| Ejection fraction decreased | 2 | 0.61 ( 0.15 - 2.43 ) | 0.61 ( 0.5 ) | 0.61 ( 0.19 ) | -0.72 ( -2.38 ) |
| Pericardial effusion malignant | 2 | 60.26 ( 14.86 - 244.34 ) | 60.26 ( 114.26 ) | 59.09 ( 18.32 ) | 5.88 ( 4.19 ) |
| Angina pectoris | 2 | 0.4 ( 0.1 - 1.58 ) | 0.4 ( 1.85 ) | 0.4 ( 0.12 ) | -1.34 ( -3.01 ) |
| Ischaemic cardiomyopathy | 2 | 5.22 ( 1.3 - 20.89 ) | 5.22 ( 6.81 ) | 5.21 ( 1.63 ) | 2.38 ( 0.71 ) |
| Myocardial necrosis marker increased | 2 | 4.77 ( 1.19 - 19.1 ) | 4.77 ( 5.95 ) | 4.76 ( 1.49 ) | 2.25 ( 0.58 ) |
| Electrocardiogram qt prolonged | 2 | 0.25 ( 0.06 - 1.02 ) | 0.25 ( 4.38 ) | 0.25 ( 0.08 ) | -1.98 ( -3.64 ) |
| Atrial thrombosis | 2 | 4.38 ( 1.09 - 17.53 ) | 4.38 ( 5.21 ) | 4.37 ( 1.37 ) | 2.13 ( 0.46 ) |
| Bradycardia | 2 | 0.19 ( 0.05 - 0.75 ) | 0.19 ( 7.08 ) | 0.19 ( 0.06 ) | -2.42 ( -4.09 ) |
| Aortic valve incompetence | 2 | 3.32 ( 0.83 - 13.29 ) | 3.32 ( 3.24 ) | 3.32 ( 1.04 ) | 1.73 ( 0.06 ) |
| Ventricular hypokinesia | 2 | 3.46 ( 0.86 - 13.84 ) | 3.46 ( 3.49 ) | 3.46 ( 1.08 ) | 1.79 ( 0.12 ) |
| Mitral valve incompetence | 2 | 1.22 ( 0.3 - 4.86 ) | 1.22 ( 0.08 ) | 1.22 ( 0.38 ) | 0.28 ( -1.39 ) |
| Coronary artery insufficiency | 1 | 23 ( 3.22 - 164.52 ) | 23 ( 20.88 ) | 22.83 ( 4.4 ) | 4.51 ( 2.46 ) |
| Prinzmetal angina | 1 | 4.95 ( 0.7 - 35.18 ) | 4.95 ( 3.14 ) | 4.94 ( 0.96 ) | 2.3 ( 0.26 ) |
| Cardiopulmonary failure | 1 | 1.53 ( 0.22 - 10.86 ) | 1.53 ( 0.18 ) | 1.53 ( 0.3 ) | 0.61 ( -1.43 ) |
| Cardiovascular insufficiency | 1 | 3.1 ( 0.44 - 22.03 ) | 3.1 ( 1.42 ) | 3.1 ( 0.6 ) | 1.63 ( -0.41 ) |
| Sinus bradycardia | 1 | 0.56 ( 0.08 - 3.97 ) | 0.56 ( 0.35 ) | 0.56 ( 0.11 ) | -0.84 ( -2.88 ) |
| Ventricular dysfunction | 1 | 3.33 ( 0.47 - 23.66 ) | 3.33 ( 1.63 ) | 3.33 ( 0.64 ) | 1.73 ( -0.31 ) |
| Nodal rhythm | 1 | 4.85 ( 0.68 - 34.5 ) | 4.85 ( 3.05 ) | 4.85 ( 0.94 ) | 2.28 ( 0.23 ) |
| Coronary artery occlusion | 1 | 0.49 ( 0.07 - 3.5 ) | 0.49 ( 0.52 ) | 0.49 ( 0.1 ) | -1.02 ( -3.06 ) |
| Nodal arrhythmia | 1 | 8.63 ( 1.21 - 61.47 ) | 8.63 ( 6.73 ) | 8.61 ( 1.67 ) | 3.11 ( 1.06 ) |
| Trifascicular block | 1 | 39.64 ( 5.51 - 285.11 ) | 39.64 ( 37.18 ) | 39.14 ( 7.51 ) | 5.29 ( 3.22 ) |
| Tachycardia induced cardiomyopathy | 1 | 27.9 ( 3.89 - 199.87 ) | 27.9 ( 25.69 ) | 27.65 ( 5.32 ) | 4.79 ( 2.73 ) |
| Autoimmune pericarditis | 1 | 251.08 ( 32.64 - 1931.17 ) | 251.06 ( 229.91 ) | 231.83 ( 42.05 ) | 7.86 ( 5.68 ) |
| Kounis syndrome | 1 | 1.85 ( 0.26 - 13.12 ) | 1.85 ( 0.39 ) | 1.85 ( 0.36 ) | 0.88 ( -1.16 ) |
| Cardiac hypertrophy | 1 | 5.37 ( 0.76 - 38.2 ) | 5.37 ( 3.55 ) | 5.36 ( 1.04 ) | 2.42 ( 0.38 ) |
| Cardiomegaly | 1 | 0.55 ( 0.08 - 3.94 ) | 0.55 ( 0.36 ) | 0.55 ( 0.11 ) | -0.85 ( -2.89 ) |
| Pericardial disease | 1 | 17.72 ( 2.48 - 126.56 ) | 17.72 ( 15.69 ) | 17.62 ( 3.4 ) | 4.14 ( 2.09 ) |
| Endocarditis | 1 | 0.96 ( 0.14 - 6.83 ) | 0.96 ( 0 ) | 0.96 ( 0.19 ) | -0.06 ( -2.1 ) |
| Cardiac failure chronic | 1 | 0.95 ( 0.13 - 6.72 ) | 0.95 ( 0 ) | 0.95 ( 0.18 ) | -0.08 ( -2.12 ) |
| Ventricular extrasystoles | 1 | 0.6 ( 0.09 - 4.29 ) | 0.6 ( 0.26 ) | 0.6 ( 0.12 ) | -0.73 ( -2.77 ) |
| Pulmonary valve incompetence | 1 | 5.02 ( 0.71 - 35.71 ) | 5.02 ( 3.22 ) | 5.01 ( 0.97 ) | 2.33 ( 0.28 ) |
| Arteriospasm coronary | 1 | 1.35 ( 0.19 - 9.59 ) | 1.35 ( 0.09 ) | 1.35 ( 0.26 ) | 0.43 ( -1.61 ) |
| Cardiorenal syndrome | 1 | 6.56 ( 0.92 - 46.7 ) | 6.56 ( 4.71 ) | 6.55 ( 1.27 ) | 2.71 ( 0.67 ) |
| Bradyarrhythmia | 1 | 3.55 ( 0.5 - 25.25 ) | 3.55 ( 1.83 ) | 3.55 ( 0.69 ) | 1.83 ( -0.22 ) |
| Myocardial ischaemia | 1 | 0.62 ( 0.09 - 4.39 ) | 0.62 ( 0.23 ) | 0.62 ( 0.12 ) | -0.69 ( -2.73 ) |
| Left ventricular failure | 1 | 1.51 ( 0.21 - 10.7 ) | 1.51 ( 0.17 ) | 1.51 ( 0.29 ) | 0.59 ( -1.45 ) |
| Arrhythmia supraventricular | 1 | 5.9 ( 0.83 - 41.94 ) | 5.9 ( 4.06 ) | 5.89 ( 1.14 ) | 2.56 ( 0.51 ) |
| Palpitations | 1 | 0.04 ( 0.01 - 0.3 ) | 0.04 ( 21.81 ) | 0.04 ( 0.01 ) | -4.57 ( -6.61 ) |
| Dilated cardiomyopathy | 1 | 5.66 ( 0.8 - 40.28 ) | 5.66 ( 3.83 ) | 5.65 ( 1.1 ) | 2.5 ( 0.46 ) |
| Electrocardiogram qrs complex prolonged | 1 | 0.96 ( 0.14 - 6.85 ) | 0.96 ( 0 ) | 0.96 ( 0.19 ) | -0.05 ( -2.09 ) |

Abbreviation: ROR, reporting odds ratio; PRR, proportional reporting ratio; EBGM, empirical Bayesian geometric mean; EBGM05, the lower limit of the 95% CI of EBGM; IC, information component; IC025, the lower limit of the 95% CI of the IC; CI, confidence interval; PT, preferred term.

**Supplementary Table S11:**

**Signal strength of cardiac adverse events associated with dual ICIs in kidney cancer patients in the FAERS database**

| PT | Numbers | ROR(95%Cl) | PRR(X²) | EBGM(EBGM05) | IC(IC025) |
| --- | --- | --- | --- | --- | --- |
| Myocarditis | 55 | 39.16 ( 30 - 51.1 ) | 38.88 ( 2015.27 ) | 38.6 ( 30.89 ) | 5.27 ( 4.88 ) |
| Atrial fibrillation | 24 | 2.09 ( 1.4 - 3.12 ) | 2.09 ( 13.65 ) | 2.09 ( 1.49 ) | 1.06 ( 0.48 ) |
| Cardiac failure | 18 | 1.91 ( 1.2 - 3.03 ) | 1.91 ( 7.75 ) | 1.91 ( 1.29 ) | 0.93 ( 0.27 ) |
| Immune-mediated myocarditis | 17 | 93.54 ( 57.88 - 151.18 ) | 93.33 ( 1525.91 ) | 91.73 ( 61.38 ) | 6.52 ( 5.83 ) |
| Cardiac arrest | 14 | 1.7 ( 1.01 - 2.88 ) | 1.7 ( 4.06 ) | 1.7 ( 1.1 ) | 0.77 ( 0.02 ) |
| Acute myocardial infarction | 10 | 3.27 ( 1.76 - 6.08 ) | 3.27 ( 15.73 ) | 3.27 ( 1.94 ) | 1.71 ( 0.84 ) |
| Pericardial effusion | 9 | 3.46 ( 1.8 - 6.65 ) | 3.45 ( 15.68 ) | 3.45 ( 2 ) | 1.79 ( 0.87 ) |
| Cardiomyopathy | 9 | 6.12 ( 3.18 - 11.77 ) | 6.11 ( 38.46 ) | 6.11 ( 3.53 ) | 2.61 ( 1.7 ) |
| Tachycardia | 8 | 0.8 ( 0.4 - 1.61 ) | 0.8 ( 0.39 ) | 0.8 ( 0.45 ) | -0.32 ( -1.28 ) |
| Troponin increased | 8 | 9.62 ( 4.81 - 19.25 ) | 9.61 ( 61.6 ) | 9.59 ( 5.37 ) | 3.26 ( 2.3 ) |
| Myocardial infarction | 7 | 0.47 ( 0.22 - 0.98 ) | 0.47 ( 4.25 ) | 0.47 ( 0.25 ) | -1.1 ( -2.12 ) |
| Cardiac disorder | 7 | 0.69 ( 0.33 - 1.44 ) | 0.69 ( 1 ) | 0.69 ( 0.37 ) | -0.54 ( -1.56 ) |
| Atrioventricular block complete | 6 | 8.76 ( 3.93 - 19.52 ) | 8.75 ( 41.14 ) | 8.74 ( 4.47 ) | 3.13 ( 2.04 ) |
| Pericarditis | 6 | 2.85 ( 1.28 - 6.34 ) | 2.84 ( 7.17 ) | 2.84 ( 1.45 ) | 1.51 ( 0.42 ) |
| Bradycardia | 5 | 0.82 ( 0.34 - 1.97 ) | 0.82 ( 0.2 ) | 0.82 ( 0.39 ) | -0.28 ( -1.46 ) |
| Arrhythmia | 4 | 0.79 ( 0.3 - 2.1 ) | 0.79 ( 0.23 ) | 0.79 ( 0.35 ) | -0.34 ( -1.63 ) |
| Cardiogenic shock | 4 | 2.43 ( 0.91 - 6.48 ) | 2.43 ( 3.37 ) | 2.43 ( 1.07 ) | 1.28 ( -0.01 ) |
| Cardiovascular disorder | 4 | 2.45 ( 0.92 - 6.53 ) | 2.45 ( 3.43 ) | 2.45 ( 1.08 ) | 1.29 ( 0 ) |
| Electrocardiogram qt prolonged | 4 | 0.89 ( 0.34 - 2.38 ) | 0.89 ( 0.05 ) | 0.89 ( 0.39 ) | -0.16 ( -1.45 ) |
| Cardiac failure congestive | 4 | 0.52 ( 0.19 - 1.38 ) | 0.52 ( 1.78 ) | 0.52 ( 0.23 ) | -0.95 ( -2.24 ) |
| Atrial flutter | 3 | 3.42 ( 1.1 - 10.6 ) | 3.42 ( 5.12 ) | 3.41 ( 1.32 ) | 1.77 ( 0.33 ) |
| Troponin i increased | 3 | 16.6 ( 5.34 - 51.56 ) | 16.59 ( 43.82 ) | 16.54 ( 6.41 ) | 4.05 ( 2.6 ) |
| Ventricular tachycardia | 3 | 1.84 ( 0.59 - 5.7 ) | 1.84 ( 1.14 ) | 1.84 ( 0.71 ) | 0.88 ( -0.57 ) |
| Cardiac tamponade | 3 | 5.41 ( 1.75 - 16.8 ) | 5.41 ( 10.78 ) | 5.41 ( 2.1 ) | 2.44 ( 0.99 ) |
| Ejection fraction decreased | 2 | 1.07 ( 0.27 - 4.27 ) | 1.07 ( 0.01 ) | 1.07 ( 0.34 ) | 0.1 ( -1.57 ) |
| Stress cardiomyopathy | 2 | 2.66 ( 0.66 - 10.64 ) | 2.66 ( 2.07 ) | 2.66 ( 0.83 ) | 1.41 ( -0.26 ) |
| Supraventricular tachycardia | 2 | 2.09 ( 0.52 - 8.36 ) | 2.09 ( 1.13 ) | 2.09 ( 0.65 ) | 1.06 ( -0.6 ) |
| Sinus tachycardia | 2 | 1.43 ( 0.36 - 5.7 ) | 1.43 ( 0.25 ) | 1.43 ( 0.45 ) | 0.51 ( -1.15 ) |
| Coronary artery stenosis | 2 | 5.23 ( 1.31 - 20.91 ) | 5.22 ( 6.83 ) | 5.22 ( 1.64 ) | 2.38 ( 0.72 ) |
| Cardiac dysfunction | 2 | 3.58 ( 0.89 - 14.31 ) | 3.57 ( 3.71 ) | 3.57 ( 1.12 ) | 1.84 ( 0.17 ) |
| Troponin t increased | 2 | 14.06 ( 3.51 - 56.33 ) | 14.06 ( 24.19 ) | 14.02 ( 4.39 ) | 3.81 ( 2.14 ) |
| Autoimmune myocarditis | 2 | 56.62 ( 14.05 - 228.11 ) | 56.6 ( 108.08 ) | 56.01 ( 17.45 ) | 5.81 ( 4.13 ) |
| Cardiac flutter | 1 | 1.32 ( 0.19 - 9.41 ) | 1.32 ( 0.08 ) | 1.32 ( 0.26 ) | 0.41 ( -1.64 ) |
| Ventricular extrasystoles | 1 | 1.06 ( 0.15 - 7.53 ) | 1.06 ( 0 ) | 1.06 ( 0.21 ) | 0.08 ( -1.96 ) |
| Ventricular dysfunction | 1 | 5.85 ( 0.82 - 41.57 ) | 5.85 ( 4.01 ) | 5.84 ( 1.13 ) | 2.55 ( 0.5 ) |
| Cardiovascular insufficiency | 1 | 5.45 ( 0.77 - 38.7 ) | 5.44 ( 3.62 ) | 5.44 ( 1.05 ) | 2.44 ( 0.4 ) |
| Pulseless electrical activity | 1 | 1.87 ( 0.26 - 13.29 ) | 1.87 ( 0.41 ) | 1.87 ( 0.36 ) | 0.9 ( -1.14 ) |
| Atrioventricular block second degree | 1 | 3.3 ( 0.47 - 23.47 ) | 3.3 ( 1.61 ) | 3.3 ( 0.64 ) | 1.72 ( -0.32 ) |
| Myocardial ischaemia | 1 | 1.09 ( 0.15 - 7.72 ) | 1.09 ( 0.01 ) | 1.09 ( 0.21 ) | 0.12 ( -1.92 ) |
| Aortic valve disease | 1 | 5.93 ( 0.83 - 42.13 ) | 5.93 ( 4.09 ) | 5.92 ( 1.15 ) | 2.57 ( 0.52 ) |
| Bundle branch block left | 1 | 2.48 ( 0.35 - 17.59 ) | 2.48 ( 0.88 ) | 2.48 ( 0.48 ) | 1.31 ( -0.73 ) |
| Sinus bradycardia | 1 | 0.98 ( 0.14 - 6.97 ) | 0.98 ( 0 ) | 0.98 ( 0.19 ) | -0.03 ( -2.07 ) |
| Cardio-respiratory arrest | 1 | 0.24 ( 0.03 - 1.7 ) | 0.24 ( 2.41 ) | 0.24 ( 0.05 ) | -2.06 ( -4.1 ) |
| Cardiac failure acute | 1 | 1.19 ( 0.17 - 8.47 ) | 1.19 ( 0.03 ) | 1.19 ( 0.23 ) | 0.25 ( -1.79 ) |
| Angina pectoris | 1 | 0.35 ( 0.05 - 2.46 ) | 0.35 ( 1.23 ) | 0.35 ( 0.07 ) | -1.53 ( -3.57 ) |
| Cardiac hypertrophy | 1 | 9.43 ( 1.33 - 67.11 ) | 9.43 ( 7.53 ) | 9.42 ( 1.82 ) | 3.24 ( 1.19 ) |
| Ejection fraction abnormal | 1 | 5.5 ( 0.77 - 39.06 ) | 5.5 ( 3.67 ) | 5.49 ( 1.06 ) | 2.46 ( 0.41 ) |
| Ventricular tachyarrhythmia | 1 | 23.52 ( 3.3 - 167.76 ) | 23.52 ( 21.47 ) | 23.42 ( 4.53 ) | 4.55 ( 2.5 ) |
| Palpitations | 1 | 0.07 ( 0.01 - 0.52 ) | 0.07 ( 11.61 ) | 0.07 ( 0.01 ) | -3.76 ( -5.8 ) |
| Bundle branch block right | 1 | 2.5 ( 0.35 - 17.74 ) | 2.5 ( 0.9 ) | 2.5 ( 0.48 ) | 1.32 ( -0.72 ) |
| Atrioventricular block | 1 | 1.2 ( 0.17 - 8.53 ) | 1.2 ( 0.03 ) | 1.2 ( 0.23 ) | 0.27 ( -1.78 ) |
| Troponin abnormal | 1 | 59.47 ( 8.28 - 426.93 ) | 59.46 ( 56.84 ) | 58.81 ( 11.3 ) | 5.88 ( 3.82 ) |

Abbreviation: ROR, reporting odds ratio; PRR, proportional reporting ratio; EBGM, empirical Bayesian geometric mean; EBGM05, the lower limit of the 95% CI of EBGM; IC, information component; IC025, the lower limit of the 95% CI of the IC; CI, confidence interval; PT, preferred term.

**Supplementary Table S12:**

**Time to Onset of Cardiovascular Events After Dual ICIs**

| strategy | 25th percentile (days) | Median(days) | 75th percentile (days) |
| --- | --- | --- | --- |
| Acute myocardial infarction | 27.75 | 52 | 89.5 |
| Angina pectoris | 11 | 64 | 86 |
| Arrhythmia | 8.5 | 38 | 98 |
| Atrial fibrillation | 15 | 33.5 | 76 |
| Atrial flutter | 7 | 18.5 | 46.5 |
| Atrioventricular block | 12 | 25 | 39 |
| Atrioventricular block complete | 7.75 | 17 | 21 |
| Bradycardia | 14.5 | 32.5 | 74.75 |
| Cardiac arrest | 14 | 24 | 77 |
| Cardiac failure | 20.5 | 63 | 93 |
| Cardiac failure acute | 15.5 | 25 | 49.5 |
| Cardiac failure congestive | 16 | 46 | 109 |
| Cardiac tamponade | 12 | 35 | 88 |
| Cardio-respiratory arrest | 11 | 24 | 43 |
| Cardiogenic shock | 11 | 11 | 18 |
| Cardiomyopathy | 17.5 | 71 | 222.5 |
| Electrocardiogram qt prolonged | 31.5 | 73 | 104.5 |
| Immune-mediated myocarditis | 11.75 | 20 | 28 |
| Myocardial infarction | 19.75 | 28.5 | 68.25 |
| Myocarditis | 14 | 20 | 39 |
| Palpitations | 10.25 | 18 | 65.25 |
| Pericardial effusion | 18 | 56 | 119.5 |
| Pericarditis | 41.5 | 59 | 135.5 |
| Sinus tachycardia | 13.5 | 42 | 83.5 |
| Stress cardiomyopathy | 8 | 27 | 53 |
| Supraventricular tachycardia | 6.5 | 19 | 36.5 |
| Tachycardia | 13 | 28 | 80 |
| Troponin i increased | 19 | 37.5 | 45 |
| Troponin increased | 20.25 | 30.5 | 45.25 |
| Ventricular tachycardia | 14 | 16 | 39 |

**Supplementary Figure S1:**

**co-reported Conditions in Patients with Myocarditis**


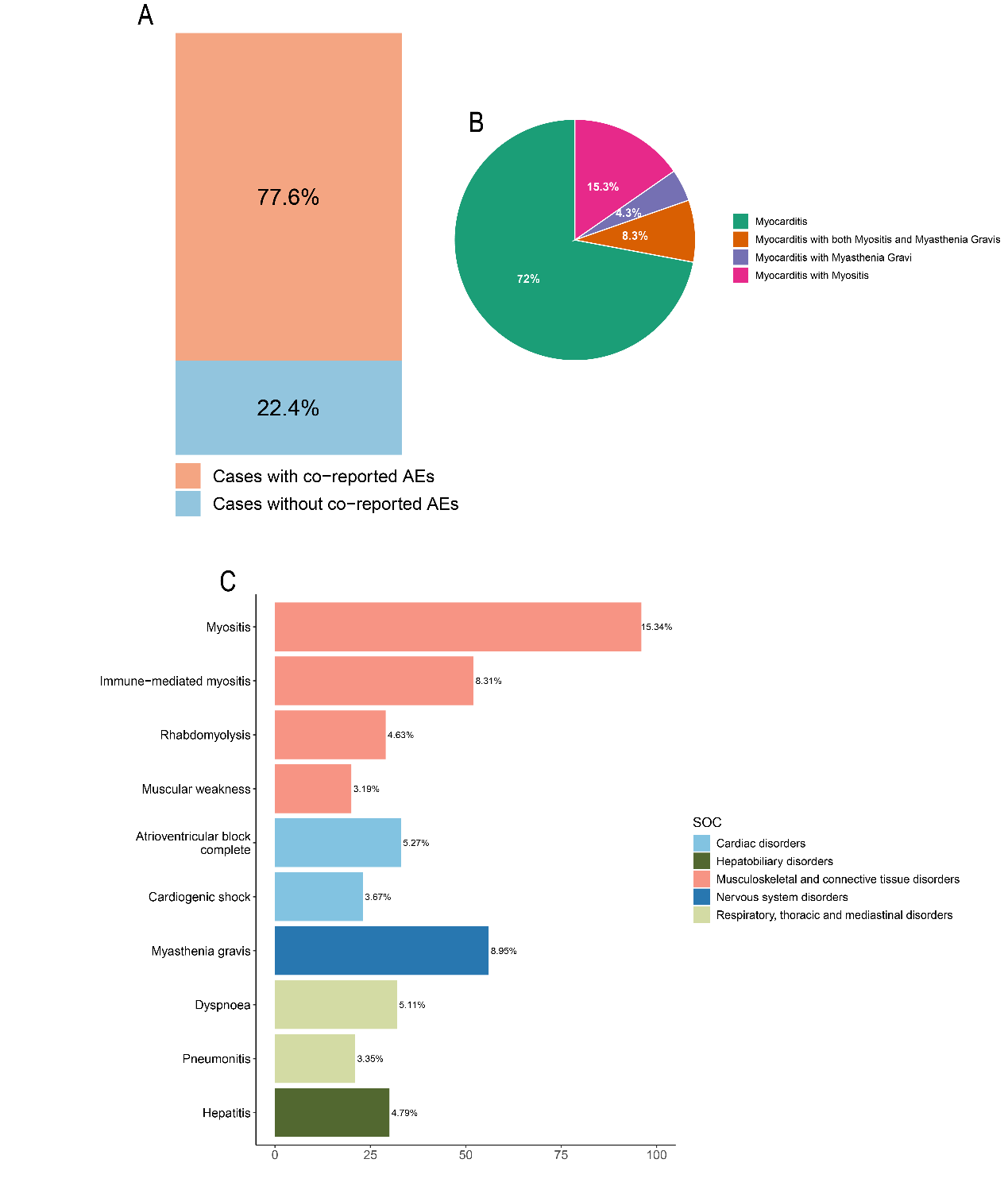


A) Bar plot shows the proportion of cases with and without co-reported adverse events incases with Myocarditis.

B) The pie chart shows the proportions of myocarditis patients with co-reported myositis and/or myasthenia gravis. C) The bar chart displays the statistical data for the most common co-reported adverse event PTs. The colors represent the corresponding SOC for each PT. The percentages marked in the chart indicate the proportion of cases with a particular adverse event relative to the total number of Myocarditis adverse event cases.

**Supplementary Figure S2:**

**Co-reported Conditions Among Patients With Atrial Fibrillation, Myocardial Infarction or Cardiac Failure**


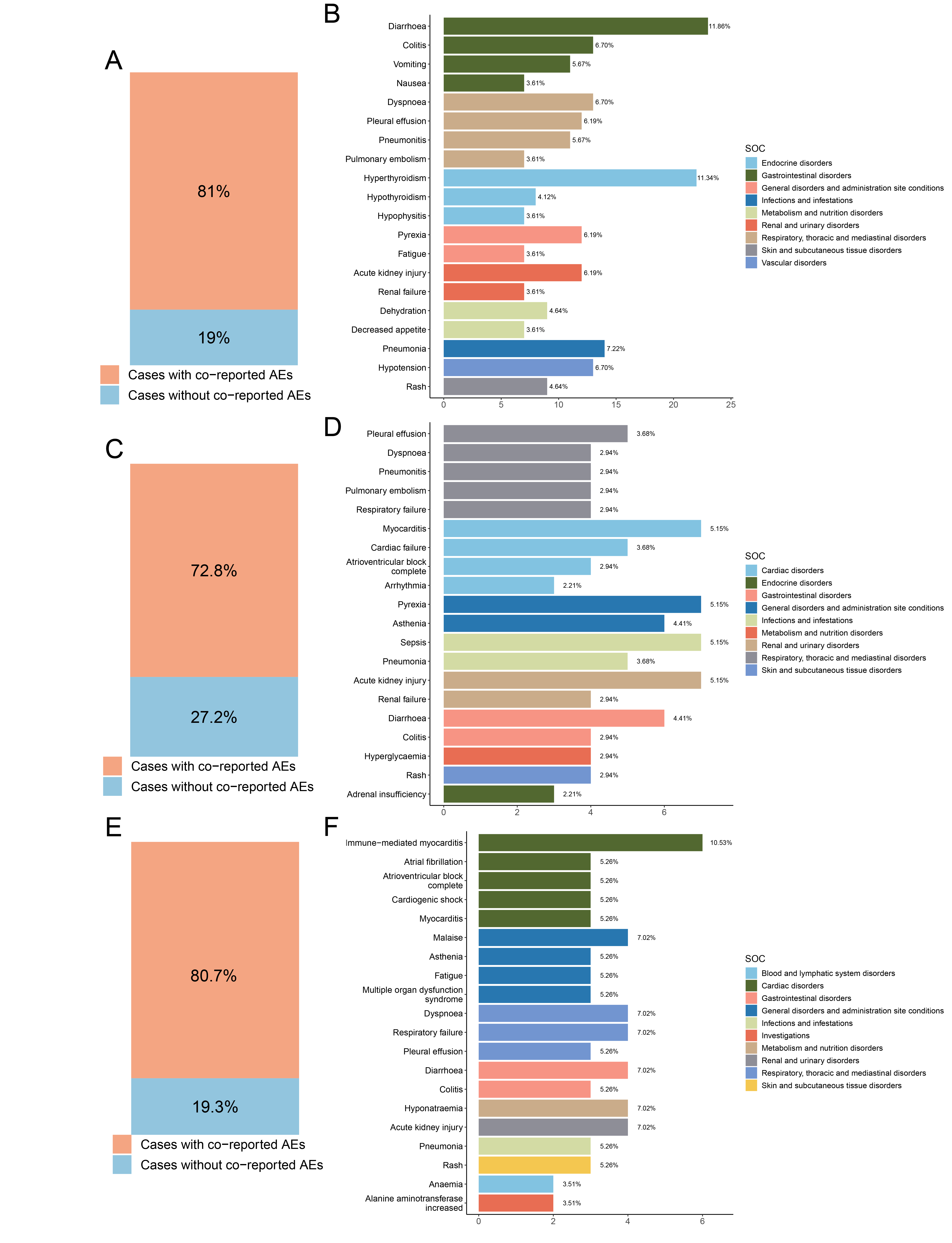


A) Bar plot shows the proportion of cases with and without co-reported adverse events incases with Atrial Fibrillation. B) Bar chart displays commonly co-reported adverse event PTs with atrial fibrillation. Colors represent SOC. Percentages show proportion of specific events to total atrial fibrillation cases. C) Bar plot shows the proportion of cases with and without co-reported adverse events incases with Myocardial Infarction. D) Bar chart displays commonly co-reported adverse event PTs with Myocardial Infarction. Colors represent SOC. Percentages show proportion of specific events to total Myocardial Infarction cases. E) Bar plot shows the proportion of cases with and without co-reported adverse events incases with Cardiac Failure. F) Bar chart displays commonly co-reported adverse event PTs with Cardiac Failure. Colors represent SOC. Percentages show proportion of specific events to total Cardiac Failure cases.

**Supplementary Figure S3:**

**Comparison of cardiac immune-related adverse events between dual and single immune checkpoint inhibitor therapies in the FAERS database.**


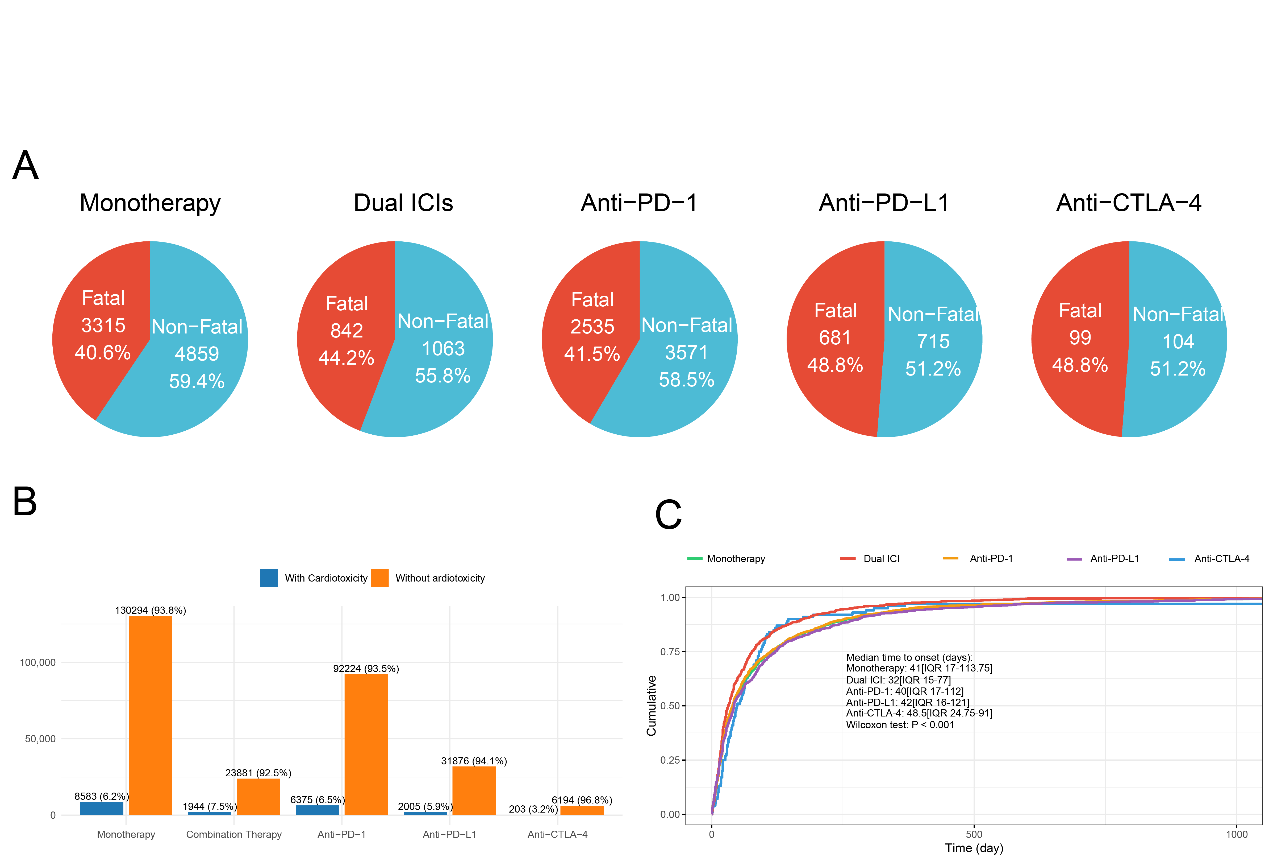


A) The pie chart shows the percentage of fatal and non-fatal outcomes associated with different ICI treatment strategies among patients with cardiac adverse events. B) Reported cases of cardiac adverse events and total reports following ICI therapy under different treatment strategies. C) Cumulative distribution curves showing the time to onset of different ICI treatment strategies among patients with cardiac adverse events.
